# Supplementary material for: Persistent Health and Cognitive Impairments up to Four Years Post-COVID-19 in Young Students: The Impact of Virus Variants and Vaccination Timing
Source: Biomedicines. 2024 Dec 30;13(1):69. doi: 10.3390/biomedicines13010069 (PMC11760454; doi:10.3390/biomedicines13010069)
Supplement: Supplementary file 1 [file biomedicines-13-00069-s001.zip › biomedicines-3333929-supplementary.pdf]

Supplements

Ashkan Latifi and Jaroslav Flegr – Persistent Health and Cognitive Impairments Up to Four Years Post-COVID-19 in Young Students: The Impact of Virus Variants and Vaccination Timing

Contents

Table S1- Distribution of data for the levels of ordinal variables 3

Table S2- Correlations between health and performance-related variables and COVID-related variables controlled for age, sex, and survey year- Women 7

Table S3- P-values of the correlations between health and performance-related variables and COVID-related variables controlled for age, sex, and survey year- Women 9

Table S4- Correlations between health and performance-related variables and COVID-related variables controlled for age, sex, and survey year- Men 11

Table S5- P-values of the correlations between health and performance-related variables and COVID-related variables controlled for age, sex, and survey year- Men 13

Table S6- Correlations between health and performance-related variables and COVID-related variables controlled for age, sex, survey year, and SARS-CoV-2 variants- All subjects 15

Table S7- P-values of the correlations between health and performance-related variables and COVID-related variables controlled for age, sex, survey year, and SARS-CoV-2 variants- All subjects 17

Table S8- Correlations between health and performance-related variables and COVID-related variables controlled for age, sex, and survey year- All Subjects beyond 24 months post-infection . 19

Table S9- P-values of the correlations between health and performance-related variables and COVID-related variables controlled for age, sex, and survey year- All Subjects beyond 24 months post-infection 21

Table S10- Correlations between health and performance-related variables and COVID-related variables controlled for age, sex, and survey year- Women beyond 24 months post-infection 23

Table S11- P-values of the correlations between health and performance-related variables and COVID-related variables controlled for age, sex, and survey year- Women beyond 24 months post-infection 25

Table S12- Correlations between health and performance-related variables and COVID-related variables controlled for age, sex, and survey year- Men beyond 24 months post-infection 27

Table S13- P-values of the correlations between health and performance-related variables and COVID-related variables controlled for age, sex, and survey year- Men beyond 24 months post-infection 29

Table S14- Correlations between health and performance-related variables and COVID-related variables controlled for age, sex, and survey year- all subjects with at least 36 months elapsed since infection 31

Table S15- P-values of the correlations between health and performance-related variables and COVID-related variables controlled for age, sex, and survey year- all subjects with at least 36 months elapsed since infection 33

Table S16- Correlations between health and performance-related variables and COVID-related variables controlled for age, sex, and survey year- Women with at least 36 months elapsed since infection 35

Table S17- P-values of the correlations between health and performance-related variables and COVID-related variables controlled for age, sex, and survey year- women with at least 36 months elapsed since infection 37

Table S18- Correlations between health and performance-related variables and COVID-related variables controlled for age, sex, and survey year- men with at least 36 months elapsed since infection 39

Table S19- P-values of the correlations between health and performance-related variables and COVID-related variables controlled for age, sex, and survey year- men with at least 36 months elapsed since infection 41

Table S20- Correlations between health and performance-related variables and COVID-related variables controlled for age, sex, and survey year- All subjects 43

Table S21- P-values of the correlations between health and performance-related variables and COVID-related variables controlled for age, sex, and survey year- All subjects 45

Table S22- Correlations between health and performance-related variables and COVID-related variables controlled for age, sex, survey year, and time elapsed since infection- All subjects 47

Table S23- P-values of the correlations between health and performance-related variables and COVID-related variables controlled for age, sex, survey year, and time elapsed since infection- All subjects 49

Figure S1- Changes in health and performance-related variables with time since COVID-related variables- Women 51

Figure S2- Changes in health and performance-related variables with time since COVID-related variables- men 51

Figure S3- Changes in health and performance-related variables with time since COVID-19 beyond 24 months post-infection- related variables- women 52

Figure S4- Changes in health and performance-related variables with time since COVID-19 beyond 24 months post-infection- related variables- men 52

Figure S5- Changes in health and performance-related variables with time since COVID vaccination-related variables- women 53

Figure S6- Changes in health and performance-related variables with time since COVID vaccination-related variables- men 53

Table S1- Distribution of data for the levels of ordinal variables

Year of Surevy		2022	2023	2024					
	Women	159 (38.3%)	99 (23.9%)	157 (37.8%)					
	Men	74 (43.8%)	39 (23.1%)	56 (33.1%)					
COVID-19 Infection		No	Yes						
	Women	205 (49.4%)	210 (50.6%)						
	Men	84 (49.7%)	85 (50.3%)						
Course of COVID-19		1	2	3	4	5	NA		
	Women	17 (4.1%)	65 (15.7%)	75 (18.1%)	40 (9.6%)	3 (0.7%)	215 (51.8%)		
	Men	9 (5.3%)	29 (17.2%)	31 (18.3%)	12 (7.1%)	1 (0.6%)	87 (51.5%)		
COVID-19 Vaccination-Binary		No	Yes						
	Women	34 (8.3%)	375 (91.7%)						
	Men	7 (4.2%)	158 (95.8%)						
COVID-19 After Vaccination		No	Yes	NA					
	Women	71 (17.1%)	29 (7.0%)	315 (75.9%)					
	Men	32 (18.9%)	17 (10.1%)	120 (71.0%)					
COVID-19 Variant		Ancestral SARS-CoV-2 variant	Alpha	Delta	Omicron	Outside waves	NA		
	Women	63 (15.2%)	19 (4.6%)	30 (7.2%)	54 (13.0%)	3 (0.7%)	246 (59.3%)		
	Men	26 (15.4%)	9 (5.3%)	22 (13.0%)	13 (7.7%)	0 (0.00)	99 (58.6%)		
Allergies		1	2	3	4	5	6		
	Women	162 (39.1%)	99 (23.9%)	92 (22.2%)	35 (8.5%)	18 (4.3%)	8 (1.9%)		
	Men	86 (50.9%)	30 (17.8%)	25 (14.8%)	15 (8.9%)	9 (5.3%)	4 (2.4%)		
Skin Problems		1	2	3	4	5	6		
	Women	165 (40.0%)	93 (22.6%)	65 (15.8%)	34 (8.3%)	16 (3.9%)	39 (9.5%)		
	Men	66 (39.3%)	38 (22.6%)	24 (14.3%)	9 (5.4%)	8 (4.8%)	23 (13.7%)		
Digestive Problems		1	2	3	4	5	6		
	Women	99 (24.0%)	129 (31.3%)	101 (24.5%)	55 (13.3%)	19 (4.6%)	9 (2.2%)		
	Men	38 (22.5%)	51 (30.2%)	58 (34.3%)	11 (6.5%)	7 (4.1%)	4 (2.4%)		
Infection		1	2	3	4	5			
	Women	8 (1.9%)	201 (48.8%)	190 (46.1%)	13 (3.2%)	0 (0.00)			
	Men	4 (2.4%)	91 (53.8%)	73 (43.2%)	0 (0.00)	1 (0.6%)			
Doctor Visits		1	2	3	4				
	Women	62 (15.0%)	234 (56.8%)	112 (27.2%)	4 (1.0%)				
	Men	21 (12.5%)	101 (60.1%)	44 (26.2%)	2 (1.2%)				

Cardiovascular Problems		1	2	3	4	5	6		
	Women	388 (93.9%)	13 (3.1%)	5 (1.2%)	4 (1.0%)	2 (0.5%)	1 (0.2%)		
	Men	161 (95.3%)	7 (4.1%)	1 (0.6%)	0 (0.00)	0 (0.00)	0 (0.00)		
Low Blood Pressure		No	Yes	NA					
	Women	148 (35.7%))	197 (47.5%)	70 (16.9%)					
	Men	89 (52.7%)	35 (20.7%)	45 (26.6%)					
High Blood Pressure		No	Yes	NA					
	Women	148 (35.7%)	25 (6.0%)	242 (58.3%)					
	Men	89 (52.7%)	18 (10.7%)	62 (36.7%)					
Orthopedic Problems		1	2	3	4	5	6		
	Women	80 (19.3%)	69 (16.7%)	113 (27.3%)	79 (19.1%)	45 (10.9%)	28 (6.8%)		
	Men	48 (28.4%)	48 (28.4%)	38 (22.5%)	16 (9.5%)	14 (8.3%)	5 (3.0%)		
Metabolic Problems		1	2	3	4				
	Women	307 (75.1%)	55 (13.4%)	26 (6.4%)	21 (5.1%)				
	Men	138 (83.6%)	18 (10.9%)	6 (3.6%)	3 (1.8%)				
Neurologic Problems		1	2	3	4	5	6		
	Women	312 (75.5%)	49 (11.9%)	21 (5.1%)	16 (3.9%)	10 (2.4%)	5 (1.2%)		
	Men	139 (83.2%)	13 (7.8%)	7 (4.2%)	4 (2.4%)	2 (1.2%)	2 (1.2%)		
Headache		1	2	3	4	5	6		
	Women	15 (3.6%)	31 (7.5%)	158 (38.2%)	130 (31.4%)	69 (16.7%)	11 (2.7%)		
	Men	10 (6.0%)	25 (15.0%)	75 (44.9%)	40 (24.0%)	17 (10.2%)	0 (0.00)		
Other Pain		1	2	3	4	5	6		
	Women	33 (8.0%)	72 (17.5%)	138 (33.5%)	115 (27.9%)	38 (9.2%)	16 (3.9%)		
	Men	18 (10.8%)	41 (24.7%)	53 (31.9%)	37 (22.3%)	10 (6.0%)	7 (4.2%)		
Recurrent Problems		1	2	3	4	5	6		
	Women	170 (41.4%)	89 (21.7%)	69 (16.8%)	45 (10.9%)	21 (5.1%)	17 (4.1%)		
	Men	80 (47.9%)	43 (25.7%)	18 (10.8%)	9 (5.4%)	8 (4.8%)	9 (5.4%)		
Depression		1	2	3	4	5	6		
	Women	135 (32.7%)	84 (20.3%)	84 (20.3%)	63 (15.3%)	34 (8.2%)	13 (3.1%)		
	Men	64 (38.3%)	38 (22.8%)	33 (19.8%)	23 (13.8%)	8 (4.8%)	1 (0.6%)		
Anxiety		1	2	3	4	5	6		
	Women	47 (11.4%)	48 (11.7%)	119 (28.9%)	88 (21.4%)	67 (16.3%)	43 (10.4%)		
	Men	34 (20.4%)	31 (18.6%)	46 (27.5%)	33 (19.8%)	15 (9.0%)	8 (4.8%)		
Other Psychological Problems		1	2	3	4	5	6		
	Women	212 (52.2%)	64 (15.8%)	60 (14.8%)	26 (6.2%)	22 (5.4%)	22 (5.4%)		
	Men	96 (57.8%)	37 (22.3%)	10 (6.0%)	14 (8.4%)	6 (3.6%)	3 (1.8%)		

Medication for Mental Purposes		1	2	3	4	5	6	7	NA
	Women	323 (77.8%)	28 (6.7%)	11 (2.7%)	6 (1.4%)	2 (0.5%)	1 (0.2%)	1 (0.2%)	44 (10.6%)
	Men	152 (89.9%)	7 (4.1%)	1 (0.6%)	0 (0.00)	0 (0.00)	0 (0)	0 (0.00)	9 (5.3%)
Other Medication		1	2	3	4	5	6	7	NA
	Women	254 (61.2%)	74 (17.8%)	32 (7.7%)	9 (2.2%)	6 (1.4%)	0 (0.00)	1 (0.2%)	39 (9.4%)
	Men	130 (76.9%)	18 (10.7%)	9 (5.3%)	2 (1.2%)	0 (0.00)	1 (0.6%)	0 (0.00)	9 (5.3%)
Antibiotics taken Past 3 Years		1	2	3	4	5	6		
	Women	136 (33.2%)	101 (24.6%)	72 (17.6%)	46 (11.2%)	19 (4.6%)	36 (8.8%)		
	Men	74 (44.3%)	41 (24.6%)	26 (15.6%)	17 (10.2%)	5 (3.0%)	4 (2.4%)		
Hospitalization Past 5 Years		1	2	3	4	5			
	Women	357 (87.1%)	36 (8.8%)	10 (2.4%)	3 (0.7%)	4 (0.1%)			
	Men	136 (81.9%)	23 (13.9%)	5 (3.0%)	1 (0.6%)	1 (0.6%)			
Life Expectancy		1	2	3	4	5	6		
	Women	17 (4.2%)	54 (13.4%)	178 (44.1%)	108 (26.7%)	26 (6.4%)	21 (5.2%)		
	Men	8 (4.8%)	15 (9.1%)	71 (43.0%)	52 (31.5%)	11 (6.7%)	8 (4.8%)		
Now Feeling Physically Miserable		1	2	3	4	5			
	Women	24 (5.9%)	137 (33.5%)	134 (32.8%)	97 (23.7%)	17 (4.2%)			
	Men	19 (11.2%)	66 (39.1%)	43 (25.4%)	36 (21.3%)	5 (3.0%)			
Now Feeling Physically Miserable 1		1	2	3	4	5			
	Women	28 (6.8%)	122 (29.5%)	142 (34.3%)	93 (22.5%)	29 (7.0%)			
	Men	21 (12.4%)	68 (40.2%)	44 (26.0%)	30 (17.8%)	6 (3.6%)			
Usually Feeling Physically Miserable		1	2	3	4	5			
	Women	40 (9.8%)	218 (53.6%)	103 (25.3%)	40 (9.8%)	6 (1.5%)			
	Men	33 (19.5%)	100 (59.2%)	27 (16.0%)	9 (5.3%)	0 (0.00)			
Usually Feeling Physically Miserable 1		1	2	3	4	5			
	Women	38 (9.2%)	226 (54.6%)	105 (25.4%)	39 (9.4%)	6 (1.4%)			
	Men	28 (16.6%)	107 (63.3%)	24 (14.2%)	10 (5.9%)	0 (0.00)			
Now Feeling Mentally Miserable		1	2	3	4	5			
	Women	10 (2.4%)	61 (14.9%)	127 (31.1%)	149 (36.4%)	62 (15.2%)			
	Men	6 (3.6%)	41 (24.3%)	53 (31.4%)	52 (30.8%)	17 (10.1%)			
Now Feeling Mentally Miserable 1		1	2	3	4	5			
	Women	31 (7.5%)	98 (23.7%)	113 (27.3%)	105 (25.4%)	67 (16.2%)			
	Men	21 (12.7%)	58 (35.2%)	41 (24.8%)	18 (10.9%)	27 (16.4%)			

Usually Feeling Mentally Miserable		1	2	3	4	5			
	Women	26 (6.4%)	150 (36.9%)	139 (34.2%)	73 (17.9%)	19 (4.7%)			
	Men	20 (11.8%)	80 (47.02)	48 (28.4%)	18 (10.7%)	3 (1.8%)			
Now Feeling Mentally Miserable 1		1	2	3	4	5			
	Women	25 (6.0%)	160 (38.6%)	142 (34.3%)	67 (16.2%)	20 (4.8%)			
	Men	21 (12.4%)	79 (46.7%)	46 (27.2%)	20 (11.8%)	3 (1.8%)			

Table S2- Correlations between health and performance-related variables and COVID-related variables controlled for age, sex, and survey year- Women

	Infected	Course	Months since Infection	Vaccination	Months since Vaccination	Covid after Vaccination	Ancestral SARS-CoV-2 variant	Alpha	Delta	Omicron	Age
Physical Health Issues	0.022	**0.208**	-0.002	**0.078**	0.045	0.061	0.072	-0.058	-0.001	-0.025	0.006
Mental Health Issues	-0.038	0.04	-0.052	0.059	0.025	**-0.02**	-0.042	0.001	-0.063	0.078	0.034
Fatigue	0.002	0.078	0.038	**0.106**	0.061	0.051	**0.141**	**-0.11**	**-0.108**	0.019	0.013
Intelligence	0.024	-0.074	-0.098	-0.023	0.032	0.203	**-0.149**	-0.044	0.033	**0.189**	-0.02
Memory	**0.065**	-0.015	-0.027	-0.032	0.002	0.066	-0.089	0.006	0.003	0.049	-0.036
Reactions	0.044	0.027	0.045	**-0.076**	0.009	-0.053	0.063	-0.041	0.004	-0.057	0.01
Accuracy	-0.004	-0.079	-0.028	-0.006	0.072	**0.265**	-0.016	**-0.11**	0.076	0.037	-0.048
Output Variables											
Evolutionary Biology Score	-0.013	0.020	0.057	0.026	0.077	**0.139**	0.035	0.065	0.052	**-0.120**	**-0.089**
Free-Recall Memory	**0.088**	-0.032	-0.040	0.007	-0.000	0.120	-0.045	-0.003	-0.053	0.055	-0.048
Recognition Memory	**0.083**	-0.026	-0.044	-0.001	0.001	0.126	-0.058	-0.024	-0.017	0.053	-0.036
Simple RT Duration	0.016	-0.073	0.049	**-0.069**	0.045	0.060	0.051	-0.037	-0.027	-0.027	0.045
Simple RT Precision	0.000	-0.010	-0.017	-0.056	0.038	0.009	0.020	**-0.232**	0.084	0.062	-0.010
Stroop Duration	0.028	0.037	0.050	-0.059	-0.015	-0.048	0.063	-0.036	0.065	**-0.104**	0.015
Stroop Precision	-0.023	-0.047	-0.044	0.004	**0.088**	**0.222**	0.039	**-0.111**	-0.044	0.061	-0.017
Reading Time	0.007	-0.053	0.047	-0.052	-0.028	-0.053	0.042	0.018	-0.097	-0.004	-0.021
Allergy	**0.066**	**0.098**	0.011	**0.079**	0.021	0.034	0.059	-0.016	-0.007	-0.018	0.014
Skin Problem	0.026	**0.183**	0.011	0.035	-0.018	**0.139**	0.048	**-0.134**	0.047	0.006	-0.016
Digestive Problem	0.013	**0.171**	-0.033	0.064	0.035	-0.076	0.036	0.042	-0.081	0.022	0.019
Infection	**0.153**	**0.217**	-0.034	0.002	-0.006	**0.166**	0.043	**-0.219**	0.090	0.062	-0.047
Cardiovascular Problem	0.032	-0.016	0.045	**0.085**	-0.031	-0.078	0.095	-0.079	-0.047	0.004	-0.036
Low Blood Pressure	-0.045	0.031	0.070	0.017	0.066	-0.100	0.083	0.008	-0.071	-0.043	-0.018
High Blood Pressure	-0.039	0.084	0.103	-0.001	0.118	0.161	0.009	0.100	0.069	-0.127	0.057
Orthopedic Problem	0.027	0.005	0.057	**0.066**	0.053	0.002	**0.102**	0.092	-0.075	**-0.118**	0.026
Metabolic Problem	0.052	-0.008	0.008	-0.003	0.001	0.050	-0.010	-0.047	**0.108**	-0.060	0.016
Neurologic Problem	0.052	**0.118**	-0.045	-0.051	0.034	**0.135**	0.005	-0.040	0.035	0.0121	0.035
Headache	0.010	**0.138**	0.0177	**0.104**	-0.034	0.109	0.079	-0.022	-0.030	-0.060	-0.049
Other Pain	-0.008	**0.105**	0.016	0.011	0.020	0.023	**0.102**	-0.053	0.029	-0.084	-0.064
Recurrent Problem	0.036	0.044	0.002	0.052	0.030	0.104	**0.160**	-0.060	-0.093	-0.010	0.048
Depression	0.020	0.068	-0.095	0.028	0.033	0.004	-0.077	-0.060	-0.039	**0.150**	**0.072**
Anxiety	0.004	0.055	-0.031	0.057	0.034	0.086	0.060	-0.091	**-0.122**	**0.120**	0.034
Other Psychological Problems	-0.008	0.086	0.052	0.027	0.019	-0.012	0.084	-0.035	-0.039	-0.020	-0.008
Medication for Mental Purposes	0.003	**0.167**	**-0.114**	0.045	-0.002	-0.010	-0.107	-0.013	0.017	**0.113**	0.029
Doctor Visits	**0.066**	**0.120**	**-0.127**	0.038	0.002	0.078	-0.071	-0.019	0.050	0.088	-0.026
Other Medication	-0.046	**0.152**	-0.025	**0.143**	0.009	0.068	0.011	**-0.131**	0.016	0.043	0.015

Antibiotic Taken Past 3 Years	**0.083**	**0.100**	-0.011	0.023	0.047	0.062	-0.007	-0.034	0.045	0.025	0.004
Hospitalization Past 5 Years	-0.012	0.082	0.007	0.026	0.028	-0.048	0.085	-0.029	0.000	-0.063	0.015
Life Expectancy	-0.040	0.092	0.072	**0.075**	-0.013	**-0.217**	-0.001	0.058	0.033	-0.073	-0.046
Now Feeling Physically Miserable	-0.028	0.066	-0.040	0.017	0.039	**0.151**	-0.061	-0.014	0.015	0.054	0.048
Usually Feeling Physically Miserable	-0.047	0.035	-0.073	0.044	-0.025	0.024	-0.097	0.036	0.035	0.029	0.020
Now Feeling Mentally Miserable	0.000	0.024	-0.012	-0.004	0.027	-0.058	-0.034	0.015	-0.031	0.006	0.042
Usually Feeling Mentally Miserable	**-0.090**	0.036	-0.084	**0.075**	-0.002	-0.055	-0.084	0.101	-0.035	0.044	0.021
Year	**0.394**	0.076	**0.591**	**-0.089**	**0.711**	**0.147**	**-0.165**	**-0.142**	0.013	**0.222**	0.021
Course of COVID-19	NA	NA	-0.034	-0.033	-0.003	0.129	0.012	-0.100	0.053	0.026	0.092
Months since COVID Infection	NA	-0.034	NA	0.024	**0.175**	**-0.390**	**0.467**	0.033	-0.059	**-0.509**	-0.011

Significant correlations are bolded. The p-values that remained or turned significant after the application of the Benjamini-Hochberg correction for multiple testing (with FDR set at 0.1) are underlined.

Table S3- P-values of the correlations between health and performance-related variables and COVID-related variables controlled for age, sex, and survey year- Women

	Infected	Course	Months since Infection	Vaccination	Months since Vaccination	Covid after Vaccination	Ancestral SARS-CoV-2 variant	Alpha	Delta	Omicron	Age
Physical Health Issues	0.512	0.00	0.966	0.018	0.284	0.376	0.165	0.268	0.981	0.627	0.847
Mental Health Issues	0.243	0.403	0.321	0.077	0.551	0.77	0.419	0.979	0.224	0.137	0.306
Fatigue	0.95	0.104	0.461	0.001	0.153	0.458	0.007	0.035	0.038	0.719	0.701
Intelligence	0.472	0.125	0.061	0.498	0.457	0.003	0.005	0.407	0.534	0.00	0.538
Memory	0.048	0.755	0.603	0.338	0.958	0.338	0.09	0.909	0.952	0.345	0.278
Reactions	0.183	0.579	0.385	0.021	0.823	0.441	0.225	0.429	0.934	0.277	0.771
Accuracy	0.898	0.099	0.588	0.856	0.089	0.00	0.756	0.034	0.146	0.481	0.143
Output Variables											
Evolutionary Biology Score	0.688	0.668	0.273	0.433	0.069	0.042	0.499	0.21	0.315	0.022	0.007
Free-Recall Memory	0.01	0.518	0.452	0.839	0.985	0.088	0.406	0.942	0.327	0.303	0.157
Recognition Memory	0.014	0.599	0.416	0.977	0.971	0.07	0.281	0.655	0.753	0.32	0.288
Simple RT Duration	0.609	0.123	0.341	0.035	0.284	0.377	0.326	0.474	0.593	0.599	0.168
Simple RT Precision	1	0.82	0.735	0.088	0.359	0.888	0.698	0.00	0.107	0.228	0.755
Stroop Duration	0.393	0.431	0.336	0.074	0.71	0.481	0.222	0.482	0.209	0.046	0.647
Stroop Precision	0.478	0.324	0.399	0.896	0.036	0.001	0.448	0.033	0.393	0.236	0.593
Reading Time	0.83	0.267	0.36	0.112	0.509	0.439	0.411	0.718	0.06	0.932	0.514
Allergy	0.044	0.04	0.823	0.017	0.62	0.612	0.254	0.752	0.892	0.726	0.663
Skin Problem	0.42	0.00	0.823	0.284	0.668	0.042	0.356	0.01	0.364	0.905	0.622
Digestive Problem	0.687	0.00	0.516	0.053	0.408	0.267	0.481	0.42	0.118	0.662	0.559
Infection	0.00	0.00	0.509	0.952	0.884	0.015	0.4	0.00	0.083	0.229	0.154
Cardiovascular Problem	0.319	0.726	0.381	0.01	0.456	0.253	0.066	0.128	0.363	0.927	0.273
Low Blood Pressure	0.207	0.55	0.218	0.641	0.153	0.18	0.141	0.879	0.209	0.45	0.618
High Blood Pressure	0.448	0.254	0.192	0.983	0.075	0.137	0.909	0.204	0.381	0.107	0.259
Orthopedic Problem	0.409	0.903	0.273	0.045	0.205	0.976	0.048	0.077	0.146	0.023	0.42
Metabolic Problem	0.114	0.864	0.871	0.921	0.976	0.46	0.839	0.365	0.037	0.246	0.627
Neurologic Problem	0.115	0.013	0.385	0.122	0.419	0.047	0.915	0.443	0.496	0.816	0.287
Headache	0.748	0.004	0.733	0.002	0.422	0.109	0.128	0.662	0.553	0.249	0.133
Other Pain	0.79	0.028	0.755	0.734	0.637	0.728	0.049	0.308	0.573	0.106	0.05
Recurrent Problem	0.275	0.361	0.96	0.117	0.479	0.128	0.002	0.245	0.074	0.834	0.145
Depression	0.533	0.151	0.067	0.386	0.429	0.946	0.139	0.248	0.454	0.004	0.028
Anxiety	0.9	0.244	0.548	0.083	0.417	0.205	0.242	0.079	0.019	0.02	0.291
Other Psychological Problems	0.8	0.072	0.32	0.408	0.654	0.859	0.108	0.497	0.45	0.693	0.797
Medication for Mental Purposes	0.915	0.001	0.037	0.198	0.948	0.886	0.051	0.808	0.747	0.038	0.394
Doctor Visits	0.044	0.012	0.014	0.243	0.956	0.254	0.169	0.711	0.335	0.091	0.414

Other Medication	0.177	0.003	0.64	0.00	0.82	0.332	0.829	0.016	0.759	0.427	0.66
Antibiotic Taken Past 3 Years	0.012	0.036	0.83	0.484	0.265	0.361	0.882	0.51	0.387	0.618	0.887
Hospitalization Past 5 Years	0.705	0.086	0.89	0.421	0.509	0.479	0.103	0.571	0.993	0.223	0.642
Life Expectancy	0.227	0.055	0.163	0.026	0.75	0.002	0.971	0.26	0.52	0.162	0.164
Now Feeling Physically Miserable	0.382	0.162	0.437	0.598	0.349	0.027	0.238	0.774	0.77	0.294	0.142
Usually Feeling Physically Miserable	0.146	0.453	0.16	0.179	0.545	0.724	0.062	0.484	0.494	0.578	0.532
Now Feeling Mentally Miserable	0.983	0.609	0.807	0.892	0.519	0.392	0.503	0.761	0.543	0.903	0.193
Usually Feeling Mentally Miserable	0.006	0.44	0.104	0.023	0.959	0.417	0.105	0.051	0.498	0.396	0.518
Year	0.00	0.109	0.00	0.007	0.00	0.03	0.001	0.006	0.789	0.00	0.505
Course of COVID-19	NA	NA	0.506	0.489	0.961	0.058	0.814	0.056	0.304	0.616	0.052
Months since COVID Infection	NA	0.506	NA	0.646	0.01	0.00	0.00	0.52	0.251	0.00	0.832

Table S4- Correlations between health and performance-related variables and COVID-related variables controlled for age, sex, and survey year- Men

	Infected	Course	Months since Infection	Vaccination	Months since Vaccination	Covid after Vaccination	Ancestral SARS-CoV-2 variant	Alpha	Delta	Omicron	Age
Physical Health Issues	-0.019	**0.220**	0.054	0.012	-0.053	-0.069	-0.021	0.014	0.043	-0.038	0.020
Mental Health Issues	-0.075	0.108	-0.033	0.042	-0.107	0.002	-0.099	0.065	0.082	-0.033	0.036
Fatigue	**-0.154**	0.139	0.108	0.029	-0.021	-0.111	0.141	-0.105	0.013	-0.104	-0.053
Intelligence	0.009	-0.007	0.034	**-0.133**	-0.048	0.088	0.031	-0.163	-0.006	0.113	-0.024
Memory	-0.000	-0.015	-0.123	-0.066	-0.047	-0.059	**-0.166**	-0.157	**0.187**	0.117	-0.098
Reactions	-0.003	0.028	0.008	-0.073	0.048	-0.073	**0.176**	**-0.230**	-0.070	0.067	-0.053
Accuracy	-0.00	-0.090	-0.008	-0.052	-0.006	0.048	-0.041	0.008	-0.137	**0.215**	-0.089
Output Variables											
Evolutionary Biology Score	-0.019	-0.007	-0.051	0.017	0.048	0.060	-0.073	0.022	-0.083	**0.176**	**-0.130**
Free-Recall Memory	-0.038	0.029	-0.134	-0.054	-0.052	-0.058	**-0.216**	-0.146	**0.264**	0.069	-0.059
Recognition Memory	-0.013	0.005	-0.109	-0.062	-0.049	-0.065	**-0.177**	**-0.204**	**0.290**	0.063	-0.056
Simple RT Duration	-0.011	-0.057	0.027	-0.026	0.040	-0.085	0.088	-0.051	-0.057	0.004	0.003
Simple RT Precision	0.056	NA	NA	-0.007	0.061	NA	NA	NA	NA	NA	0.001
Stroop Duration	-0.013	0.044	0.010	-0.025	0.076	-0.028	0.114	-0.118	-0.071	0.047	-0.021
Stroop Precision	0.036	**-0.259**	0.059	-0.067	-0.054	-0.105	0.050	0.126	-0.126	-0.019	-0.012
Reading Time	**0.103**	0.085	0.005	-0.034	0.012	-0.114	0.086	**-0.202**	0.128	-0.090	0.037
Allergy	-0.090	**0.201**	0.020	0.026	0.017	0.031	0.005	0.031	0.079	-0.133	-0.064
Skin Problem	-0.006	0.119	0.003	-0.048	0.119	0.131	-0.037	0.090	0.003	-0.037	-0.054
Digestive Problem	-0.053	0.006	0.000	0.067	0.052	0.015	-0.078	0.028	0.044	0.019	0.041
Infection	-0.033	0.121	0.103	**0.130**	-0.048	-0.113	0.106	0.080	-0.130	-0.045	-0.078
Cardiovascular Problem	-0.036	0.089	-0.023	0.040	-0.037	0.175	-0.120	**0.220**	-0.104	0.086	-0.014
Low Blood Pressure	-0.056	-0.014	0.029	**0.140**	-0.008	-0.111	0.019	0.030	0.122	**-0.221**	0.006
High Blood Pressure	-0.094	-0.137	-0.093	0.097	0.052	0.158	-0.143	-0.142	0.101	0.173	**0.129**
Orthopedic Problem	0.027	0.148	-0.040	-0.028	-0.046	-0.004	**-0.225**	**0.168**	0.077	0.040	**0.105**
Metabolic Problem	-0.010	**0.199**	0.083	-0.073	-0.073	-0.132	0.043	0.108	-0.056	-0.082	0.032
Neurologic Problem	0.082	0.144	0.028	-0.003	0.009	-0.056	-0.016	-0.098	-0.033	0.149	-0.054
Headache	-0.047	0.064	0.042	**0.113**	-0.009	-0.119	-0.147	**0.200**	-0.001	0.011	-0.034
Other Pain	-0.053	**0.191**	0.044	-0.012	-0.033	-0.001	-0.019	0.089	-0.053	0.011	0.047
Recurrent Problem	-0.003	**0.173**	0.051	0.080	-0.098	-0.041	0.048	0.076	-0.066	-0.048	-0.011
Depression	-0.021	0.143	-0.037	-0.034	**-0.128**	-0.174	-0.070	0.042	-0.005	0.058	0.052
Anxiety	-0.026	0.101	-0.120	0.018	-0.070	0.055	**-0.182**	0.017	0.089	0.106	0.036
Other Psychological Problems	**-0.106**	**0.187**	-0.072	0.029	-0.047	-0.080	-0.120	0.122	-0.067	0.126	0.050
Medication for Mental Purposes	**-0.118**	**0.154**	0.065	0.042	0.028	-0.136	0.023	-0.054	0.092	-0.093	0.059
Doctor Visits	-0.000	**0.194**	0.025	-0.031	0.055	-0.165	0.028	**0.199**	**-0.206**	0.042	**-0.103**
Other Medication	-0.068	**0.273**	0.001	0.019	-0.013	0.125	0.021	0.088	**-0.184**	0.116	-0.019
Antibiotic Taken Past 3 Years	0.010	0.099	-0.009	-0.077	0.017	-0.045	-0.019	0.027	-0.023	0.030	-0.057

Hospitalization Past 5 Years	**0.112**	**0.176**	0.045	**-0.106**	-0.055	-0.097	-0.018	0.047	0.075	-0.112	**-0.114**
Life Expectancy	0.040	0.090	0.017	-0.091	-0.044	0.039	0.038	**-0.183**	0.115	-0.029	0.037
Now Feeling Physically Miserable	0.049	0.072	0.125	0.007	-0.111	**-0.233**	0.103	0.002	-0.058	-0.062	**0.130**
Usually Feeling Physically Miserable	-0.024	0.071	-0.018	-0.027	-0.090	-0.078	0.003	-0.131	0.045	0.055	0.046
Now Feeling Mentally Miserable	-0.060	0.086	0.053	0.002	**-0.148**	0.069	0.014	0.010	0.125	**-0.183**	0.043
Usually Feeling Mentally Miserable	-0.084	0.018	-0.020	0.084	-0.039	-0.1747	-0.046	0.155	-0.027	-0.045	0.014
Year	**0.315**	**0.178**	**0.684**	**-0.123**	**0.710**	-0.011	-0.107	-0.112	-0.066	**0.306**	0.009
Course of COVID-19	NA	NA	0.065	-0.042	-0.002	-0.147	0.084	0.125	-0.112	-0.080	0.129
Months since COVID Infection	-0.078	0.065	NA	0.086	0.067	**-0.441**	**0.485**	0.035	**-0.223**	**-0.374**	0.031

Significant correlations are bolded. The p-values that remained or turned significant after the application of the Benjamini-Hochberg correction for multiple testing (with FDR set at 0.1) are underlined.

Table S5- P-values of the correlations between health and performance-related variables and COVID-related variables controlled for age, sex, and survey year- Men

	Infected	Course	Months since Infection	Vaccination	Months since Vaccination	Covid after Vaccination	Ancestral SARS-CoV-2 variant	Alpha	Delta	Omicron	Age
Physical Health Issues	0.713	0.004	0.51	0.819	0.404	0.49	0.797	0.866	0.603	0.639	0.687
Mental Health Issues	0.149	0.155	0.685	0.419	0.096	0.983	0.229	0.432	0.318	0.684	0.485
Fatigue	0.003	0.069	0.19	0.575	0.743	0.27	0.087	0.205	0.87	0.21	0.304
Intelligence	0.851	0.924	0.678	0.013	0.454	0.381	0.706	0.05	0.939	0.176	0.648
Memory	0.994	0.843	0.14	0.206	0.467	0.56	0.047	0.06	0.025	0.16	0.06
Reactions	0.953	0.711	0.92	0.164	0.448	0.469	0.033	0.005	0.398	0.417	0.306
Accuracy	1	0.234	0.916	0.315	0.926	0.629	0.616	0.916	0.098	0.009	0.084
Output Variables											
Evolutionary Biology Score	0.705	0.92	0.535	0.737	0.449	0.551	0.378	0.787	0.315	0.033	0.012
Free-Recall Memory	0.482	0.713	0.123	0.332	0.435	0.584	0.013	0.092	0.002	0.423	0.278
Recognition Memory	0.813	0.942	0.221	0.254	0.46	0.539	0.047	0.022	0.001	0.48	0.301
Simple RT Duration	0.823	0.454	0.745	0.616	0.527	0.396	0.287	0.532	0.49	0.955	0.939
Simple RT Precision	0.278	NA	NA	0.892	0.34	NA	NA	NA	NA	NA	0.977
Stroop Duration	0.79	0.558	0.898	0.626	0.237	0.778	0.168	0.154	0.392	0.566	0.685
Stroop Precision	0.488	0.001	0.474	0.202	0.396	0.295	0.546	0.127	0.127	0.817	0.815
Reading Time	0.047	0.26	0.944	0.513	0.846	0.258	0.295	0.015	0.12	0.274	0.47
Allergy	0.083	0.008	0.802	0.62	0.785	0.756	0.946	0.702	0.34	0.107	0.212
Skin Problem	0.905	0.116	0.971	0.362	0.065	0.192	0.653	0.276	0.965	0.651	0.294
Digestive Problem	0.303	0.936	0.997	0.202	0.418	0.879	0.343	0.73	0.588	0.818	0.424
Infection	0.52	0.11	0.21	0.013	0.457	0.261	0.198	0.332	0.115	0.581	0.132
Cardiovascular Problem	0.482	0.242	0.779	0.438	0.566	0.082	0.147	0.008	0.208	0.296	0.783
Low Blood Pressure	0.355	0.87	0.751	0.023	0.909	0.34	0.839	0.75	0.195	0.019	0.92
High Blood Pressure	0.152	0.143	0.345	0.146	0.515	0.187	0.147	0.148	0.302	0.079	0.05
Orthopedic Problem	0.596	0.051	0.623	0.586	0.473	0.966	0.007	0.042	0.348	0.623	0.043
Metabolic Problem	0.838	0.009	0.315	0.167	0.263	0.188	0.598	0.193	0.497	0.323	0.539
Neurologic Problem	0.115	0.059	0.736	0.954	0.883	0.574	0.847	0.238	0.692	0.074	0.296
Headache	0.36	0.398	0.609	0.032	0.885	0.236	0.078	0.017	0.985	0.893	0.514
Other Pain	0.308	0.012	0.594	0.815	0.601	0.986	0.812	0.286	0.522	0.893	0.367
Recurrent Problem	0.953	0.024	0.535	0.128	0.129	0.681	0.562	0.363	0.428	0.559	0.827
Depression	0.686	0.059	0.652	0.519	0.048	0.084	0.397	0.606	0.949	0.484	0.315
Anxiety	0.615	0.184	0.146	0.727	0.275	0.582	0.028	0.837	0.281	0.2	0.48
Other Psychological Problems	0.044	0.014	0.385	0.582	0.467	0.424	0.149	0.141	0.421	0.129	0.332
Medication for Mental Purposes	0.027	0.048	0.442	0.431	0.663	0.188	0.779	0.519	0.274	0.269	0.266
Doctor Visits	0.99	0.011	0.758	0.551	0.392	0.1	0.732	0.016	0.013	0.61	0.047
Other Medication	0.199	0.001	0.99	0.721	0.84	0.226	0.8	0.296	0.03	0.17	0.711

Antibiotic Taken Past 3 Years	0.841	0.191	0.912	0.144	0.781	0.65	0.813	0.743	0.776	0.716	0.275
Hospitalization Past 5 Years	0.032	0.02	0.584	0.045	0.397	0.335	0.824	0.568	0.362	0.175	0.029
Life Expectancy	0.439	0.242	0.837	0.084	0.493	0.702	0.643	0.028	0.165	0.725	0.477
Now Feeling Physically Miserable	0.346	0.338	0.13	0.887	0.084	0.021	0.21	0.98	0.483	0.454	0.012
Usually Feeling Physically Miserable	0.643	0.351	0.822	0.6	0.16	0.439	0.963	0.113	0.581	0.506	0.37
Now Feeling Mentally Miserable	0.243	0.255	0.515	0.961	0.021	0.489	0.859	0.901	0.131	0.027	0.4
Usually Feeling Mentally Miserable	0.103	0.805	0.807	0.111	0.539	0.083	0.578	0.061	0.741	0.582	0.783
Year	0.00	0.019	0.00	0.018	0.00	0.904	0.191	0.174	0.418	0.00	0.848
Course of COVID-19	NA	NA	0.439	0.585	0.983	0.148	0.316	0.137	0.181	0.337	0.088
Months since COVID Infection	0.347	0.439	NA	0.311	0.503	0.00	0.00	0.668	0.007	0.00	0.7

Table S6- Correlations between health and performance-related variables and COVID-related variables controlled for age, sex, survey year, and SARS-CoV-2 variants- All subjects

	Infected	Course	Months since Infection	Vaccination	Months since Vaccination	Covid after Vaccination	Ancestral SARS-CoV-2 variant	Alpha	Delta	Omicron	Age	Sex
Physical Health Issues	0.011	**0.210**	-0.009	**0.060**	0.012	0.010	0.031	-0.026	0.009	0.000	0.011	**0.162**
Mental Health Issues	-0.047	0.066	-0.021	0.050	-0.014	-0.042	-0.017	0.021	-0.034	0.021	0.036	**0.162**
Fatigue	-0.043	**0.092**	0.009	**0.086**	0.034	0.047	**0.119**	**-0.107**	-0.048	0.065	-0.005	**0.096**
Intelligence	0.025	-0.056	0.011	-0.035	0.009	0.099	0.039	-0.068	-0.014	**0.088**	-0.020	**-0.099**
Memory	0.047	-0.008	0.006	-0.031	-0.007	-0.053	-0.052	-0.028	0.042	-0.018	-0.054	**0.111**
Reactions	0.027	0.028	0.005	**-0.079**	0.025	-0.018	0.082	**-0.098**	-0.012	0.029	-0.009	0.020
Accuracy	0.003	**-0.081**	0.004	-0.010	0.054	**0.174**	0.033	-0.070	-0.001	0.051	-0.052	**-0.095**
Output Variables												
Evolutionary Biology Score	-0.014	0.008	0.015	0.023	0.066	**0.126**	-0.036	0.050	0.017	-0.039	**-0.099**	**-0.073**
Free-Recall Memory	0.053	-0.017	-0.018	0.001	-0.011	-0.004	-0.038	-0.036	0.036	-0.016	-0.051	**0.107**
Recognition Memory	**0.059**	-0.018	-0.013	-0.005	-0.007	-0.004	-0.030	-0.068	0.064	-0.025	-0.041	**0.115**
Simple RT Duration	0.003	-0.069	0.020	**-0.060**	0.041	0.065	0.049	-0.051	-0.028	0.022	0.034	0.045
Simple RT Precision	0.009	-0.008	0.006	-0.044	0.045	0.059	**0.090**	**-0.191**	0.059	0.032	-0.006	**-0.074**
Stroop Duration	0.013	0.039	0.000	-0.054	0.010	0.009	0.048	-0.072	0.0311	-0.017	0.000	-0.002
Stroop Precision	-0.006	**-0.094**	-0.026	-0.009	0.045	**0.110**	0.060	-0.042	-0.070	0.070	-0.015	-0.007
Reading Time	0.039	-0.011	0.025	-0.053	-0.011	-0.049	0.033	-0.055	-0.008	0.006	-0.002	0.053
Allergy	0.016	**0.127**	-0.014	**0.066**	0.018	0.074	0.014	-0.001	0.031	-0.022	-0.006	**0.069**
Skin Problem	0.016	**0.163**	0.004	0.017	0.029	0.094	0.025	-0.055	0.031	-0.002	-0.029	-0.017
Digestive Problem	-0.003	**0.125**	-0.022	**0.063**	0.039	-0.059	0.005	0.040	-0.041	0.032	0.025	0.004
Infection	**0.102**	**0.189**	0.00	0.031	-0.013	0.039	**0.102**	**-0.127**	0.017	0.052	**-0.055**	**0.055**
Cardiovascular Problem	0.015	0.003	0.019	**0.075**	-0.032	-0.017	0.052	-0.011	-0.058	0.055	-0.029	0.026
Low Blood Pressure	-0.048	0.020	0.027	0.039	0.048	-0.090	0.005	0.009	0.004	-0.030	-0.014	**0.255**
High Blood Pressure	-0.059	0.001	0.049	0.037	0.090	**0.156**	-0.039	-0.003	0.077	-0.052	**0.088**	-0.034
Orthopedic Problem	0.023	0.045	0.001	0.042	0.020	0.038	-0.057	**0.104**	-0.017	-0.044	0.045	**0.159**
Metabolic Problem	0.039	0.042	0.015	-0.017	-0.019	-0.001	-0.029	-0.011	0.070	-0.068	0.021	**0.095**
Neurologic Problem	**0.061**	**0.125**	-0.020	-0.041	0.025	0.104	0.039	-0.056	0.008	0.033	0.012	**0.078**
Headache	-0.006	**0.130**	-0.003	**0.108**	-0.025	-0.007	-0.026	0.044	-0.014	-0.024	-0.043	**0.160**
Other Pain	-0.021	**0.140**	-0.016	0.001	0.002	0.066	0.031	-0.018	0.010	-0.010	-0.028	**0.088**
Recurrent Problem	0.024	0.079	-0.031	0.050	-0.013	0.103	**0.094**	-0.022	-0.068	0.068	0.029	**0.070**
Depression	0.010	**0.089**	-0.034	0.019	-0.013	-0.071	0.015	-0.023	-0.053	0.076	**0.069**	**0.081**
Anxiety	-0.006	0.070	-0.023	0.050	0.004	0.060	0.071	-0.056	-0.069	**0.108**	0.035	**0.155**
Other Psychological Problems	-0.034	**0.114**	0.019	0.024	-0.001	-0.025	0.034	0.002	-0.047	0.040	0.008	**0.072**
Medication for Mental Purposes	-0.020	**0.155**	-0.033	0.048	0.005	**-0.129**	-0.013	-0.021	0.020	0.017	0.036	**0.117**
Doctor Visits	0.046	**0.144**	-0.065	0.024	0.015	-0.050	0.002	0.051	-0.050	0.058	-0.049	-0.009
Other Medication	-0.051	**0.185**	-0.014	**0.117**	0.001	0.075	0.059	-0.067	-0.049	0.064	0.004	**0.136**

Antibiotic Taken Past 3 Years	**0.064**	**0.099**	0.004	0.004	0.034	0.012	0.012	-0.012	0.017	0.016	-0.015	**0.117**
Hospitalization Past 5 Years	0.018	**0.124**	-0.0171	-0.008	-0.000	-0.042	0.003	-0.001	0.035	-0.038	-0.028	**-0.058**
Life Expectancy	-0.015	**0.095**	0.050	0.035	-0.025	-0.103	-0.019	-0.014	0.067	-0.061	-0.020	-0.037
Now Feeling Physically Miserable	-0.006	0.073	0.008	0.017	-0.007	0.024	0.009	-0.009	-0.020	0.021	**0.073**	**0.102**
Usually Feeling Physically Miserable	-0.037	0.050	-0.033	0.033	-0.045	-0.048	-0.034	-0.006	0.022	-0.010	0.029	**0.169**
Now Feeling Mentally Miserable	-0.021	0.053	-0.006	-0.006	-0.028	-0.022	-0.042	0.009	0.026	-0.051	0.043	**0.128**
Usually Feeling Mentally Miserable	**-0.087**	0.034	-0.058	**0.070**	-0.015	**-0.136**	-0.073	**0.118**	-0.045	0.001	0.021	**0.149**
Year	**0.372**	**0.106**	**0.720**	**-0.081**	**0.706**	-0.108	0.045	**-0.117**	-0.064	**0.133**	0.019	0.048
Course of COVID-19	NA	NA	-0.020	-0.037	-0.003	0.018	0.037	-0.034	0.002	0.017	**0.101**	0.050
Months since COVID Infection	-0.012	-0.020	NA	-0.015	0.097	**-0.163**	**0.086**	0.002	0.013	**-0.153**	-0.002	-0.028

Sex (last column) was coded as 1 – men and 2 – women. Significant correlations are bolded. The p-values that remained or turned significant after the application of the Benjamini-Hochberg correction for multiple testing (with FDR set at 0.1) are underlined.

Table S7- P-values of the correlations between health and performance-related variables and COVID-related variables controlled for age, sex, survey year, and SARS-CoV-2 variants- All subjects

	Infected	Course	Months since Infection	Vaccination	Months since Vaccination	Covid after Vaccination	Ancestral SARS-CoV-2 variant	Alpha	Delta	Omicron	Age	Sex
Physical Health Issues	0.673	0.00	0.83	0.03	0.715	0.85	0.474	0.551	0.831	0.997	0.678	0.00
Mental Health Issues	0.084	0.1	0.616	0.07	0.682	0.446	0.697	0.628	0.429	0.628	0.188	0.00
Fatigue	0.116	0.022	0.834	0.002	0.326	0.395	0.006	0.014	0.273	0.136	0.835	0.001
Intelligence	0.374	0.163	0.786	0.205	0.795	0.077	0.372	0.122	0.747	0.044	0.474	0.00
Memory	0.089	0.827	0.891	0.26	0.831	0.347	0.235	0.519	0.332	0.67	0.052	0.00
Reactions	0.326	0.483	0.901	0.005	0.477	0.739	0.059	0.024	0.771	0.508	0.733	0.463
Accuracy	0.912	0.043	0.926	0.711	0.121	0.002	0.439	0.107	0.978	0.236	0.06	0.001
Output Variables												
Evolutionary Biology Score	0.602	0.835	0.719	0.394	0.062	0.024	0.409	0.252	0.692	0.37	0	0.009
Free-Recall Memory	0.065	0.683	0.685	0.972	0.75	0.942	0.402	0.422	0.421	0.714	0.079	0.00
Recognition Memory	0.041	0.657	0.765	0.839	0.828	0.933	0.51	0.135	0.163	0.586	0.149	0.00
Simple RT Duration	0.889	0.086	0.643	0.03	0.24	0.24	0.262	0.244	0.519	0.613	0.217	0.1
Simple RT Precision	0.72	0.835	0.88	0.113	0.195	0.286	0.039	0.00	0.177	0.464	0.804	0.007
Stroop Duration	0.622	0.326	0.994	0.05	0.755	0.86	0.267	0.096	0.477	0.692	0.983	0.938
Stroop Precision	0.822	0.019	0.545	0.73	0.201	0.049	0.165	0.338	0.11	0.109	0.568	0.774
Reading Time	0.157	0.766	0.566	0.055	0.755	0.373	0.442	0.205	0.852	0.877	0.931	0.055
Allergy	0.56	0.002	0.738	0.018	0.595	0.186	0.746	0.967	0.477	0.607	0.817	0.012
Skin Problem	0.566	0.00	0.919	0.536	0.405	0.092	0.566	0.208	0.469	0.963	0.282	0.521
Digestive Problem	0.89	0.002	0.615	0.024	0.261	0.285	0.899	0.356	0.343	0.465	0.353	0.874
Infection	0.00	0.00	0.999	0.257	0.701	0.482	0.019	0.004	0.697	0.229	0.048	0.047
Cardiovascular Problem	0.567	0.922	0.651	0.007	0.358	0.759	0.232	0.795	0.181	0.204	0.291	0.349
Low Blood Pressure	0.116	0.647	0.571	0.211	0.219	0.148	0.903	0.845	0.933	0.524	0.642	0.00
High Blood Pressure	0.139	0.975	0.42	0.366	0.075	0.047	0.516	0.956	0.201	0.388	0.028	0.396
Orthopedic Problem	0.396	0.256	0.965	0.127	0.554	0.49	0.191	0.017	0.696	0.306	0.099	0.00
Metabolic Problem	0.162	0.291	0.715	0.531	0.589	0.982	0.505	0.799	0.106	0.116	0.446	0.001
Neurologic Problem	0.027	0.002	0.638	0.143	0.471	0.063	0.365	0.195	0.843	0.449	0.66	0.005
Headache	0.825	0.001	0.941	0.00	0.469	0.895	0.553	0.305	0.742	0.583	0.12	0.00
Other Pain	0.447	0.00	0.711	0.945	0.94	0.237	0.473	0.676	0.812	0.815	0.303	0.001
Recurrent Problem	0.377	0.05	0.469	0.072	0.704	0.066	0.031	0.616	0.122	0.119	0.283	0.012
Depression	0.705	0.027	0.432	0.489	0.707	0.201	0.72	0.595	0.223	0.079	0.012	0.004
Anxiety	0.821	0.081	0.594	0.071	0.908	0.284	0.101	0.196	0.112	0.013	0.207	0.00
Other Psychological Problems	0.224	0.005	0.652	0.395	0.962	0.655	0.435	0.949	0.282	0.363	0.767	0.01
Medication for Mental Purposes	0.475	0.00	0.47	0.096	0.879	0.026	0.777	0.644	0.661	0.708	0.208	0.00
Doctor Visits	0.095	0.00	0.133	0.391	0.652	0.367	0.956	0.236	0.248	0.183	0.075	0.74
Other Medication	0.074	0.00	0.742	0.00	0.963	0.188	0.191	0.138	0.28	0.155	0.88	0.00

Antibiotic Taken Past 3 Years	0.022	0.013	0.91	0.863	0.325	0.821	0.782	0.773	0.696	0.705	0.582	0.00
Hospitalization Past 5 Years	0.518	0.002	0.696	0.768	0.993	0.445	0.932	0.969	0.415	0.375	0.309	0.035
Life Expectancy	0.571	0.019	0.249	0.212	0.471	0.065	0.652	0.737	0.122	0.159	0.463	0.177
Now Feeling Physically Miserable	0.813	0.066	0.843	0.54	0.838	0.657	0.833	0.835	0.64	0.631	0.008	0.00
Usually Feeling Physically Miserable	0.182	0.207	0.445	0.23	0.2	0.385	0.429	0.891	0.6	0.818	0.29	0.00
Now Feeling Mentally Miserable	0.43	0.186	0.889	0.82	0.423	0.695	0.328	0.829	0.539	0.236	0.119	0.00
Usually Feeling Mentally Miserable	0.002	0.392	0.182	0.011	0.669	0.015	0.094	0.007	0.299	0.981	0.441	0.00
Year	0	0.008	0.00	0.004	0.00	0.052	0.3	0.007	0.137	0.002	0.474	0.08
Course of COVID-19	NA	NA	0.644	0.356	0.948	0.737	0.4	0.433	0.962	0.697	0.012	0.21
Months since COVID Infection	0.767	0.644	NA	0.72	0.083	0.004	0.05	0.952	0.758	0.00	0.946	0.52

Table S8- Correlations between health and performance-related variables and COVID-related variables controlled for age, sex, and survey year- All Subjects beyond 24 months post-infection

	Infected	Course	Months since Infection	Vaccination	Months since Vaccination	Covid after Vaccination	Ancestral SARS-CoV-2 variant	Alpha	Delta	Omicron	Age	Sex
Physical Health Issues	NA	**0.234**	0.045	-0.021	-0.009	**-0.218**	0.067	0.048	0.014	-0.101	0.015	0.057
Mental Health Issues	NA	0.047	0.028	0.019	0.016	-0.173	-0.018	0.071	-0.001	-0.062	0.023	0.069
Fatigue	NA	0.097	0.041	0.106	-0.003	-0.13	0.12	-0.044	-0.095	-0.016	**0.13**	**0.248**
Intelligence	NA	0.007	**-0.193**	**-0.172**	-0.009	0.183	**-0.144**	-0.071	0.086	**0.176**	-0.024	**-0.136**
Memory	NA	0.028	-0.079	**-0.13**	0.002	0.097	-0.109	-0.113	0.079	0.065	-0.043	**0.171**
Reactions	NA	0.021	0.091	**-0.164**	0.079	-0.09	0.103	-0.092	-0.064	-0.016	0.024	0.073
Accuracy	NA	-0.034	-0.09	**-0.141**	-0.04	0.166	-0.099	-0.073	0.066	0.109	-0.097	-0.052
Output Variables												
Evolutionary Biology Score	NA	-0.002	0.02	0.025	-0.014	**0.19**	-0.053	0.069	0.057	-0.034	**-0.161**	0.012
Free-Recall Memory	NA	0.029	-0.071	-0.075	0.042	0.14	-0.089	-0.067	0.031	0.047	-0.028	**0.189**
Recognition Memory	NA	0.027	-0.076	-0.098	0.03	0.144	-0.077	**-0.151**	0.074	0.056	-0.042	**0.201**
Simple RT Duration	NA	-0.036	0.109	**-0.172**	0.166	0.004	0.097	-0.069	-0.119	0.013	0.007	0.011
Simple RT Precision	NA	0.022	-0.01	-0.078	0.067	**-0.245**	-0.109	0.056	0.09	-0.017	-0.012	-0.09
Stroop Duration	NA	0.041	0.049	-0.112	-0.005	-0.079	0.029	-0.009	-0.004	-0.032	-0.011	0.059
Stroop Precision	NA	-0.069	0.03	-0.128	-0.014	0.047	**0.165**	**-0.256**	-0.092	0.051	-0.058	-0.095
Reading Time	NA	-0.013	0.086	-0.059	0.12	0.031	0.088	-0.077	-0.014	-0.08	0.045	0.091
Allergy	NA	**0.182**	0.009	**0.16**	-0.014	-0.098	0.055	**0.175**	-0.054	-0.079	0.016	0.037
Skin Problem	NA	**0.201**	0.037	-0.008	-0.138	-0.018	0.004	0.013	0.04	-0.045	0.012	-0.067
Digestive Problem	NA	0.098	-0.056	-0.046	-0.043	-0.098	-0.014	-0.007	-0.023	0.075	0.107	-0.017
Infection	NA	**0.137**	0.01	**-0.137**	-0.005	0.165	0.066	-0.083	0.029	0.007	-0.055	0.094
Cardiovascular Problem	NA	-0.001	-0.03	0.112	**-0.189**	-0.175	-0.019	0.055	-0.03	0.032	-0.073	0.049
Low Blood Pressure	NA	-0.051	0.008	**-0.183**	0.023	-0.154	-0.032	0.047	-0.018	0.009	0.041	**0.289**
High Blood Pressure	NA	0.109	-0.108	-0.113	0.012	0.202	**-0.234**	0.096	0.159	0.073	**0.214**	0.089
Orthopedic Problem	NA	0.009	**0.203**	0.1	0.136	-0.176	**0.175**	**0.145**	-0.056	**-0.254**	0.06	0.032
Metabolic Problem	NA	0.104	-0.12	-0.096	-0.049	0.064	-0.117	-0.108	**0.175**	0.019	0.023	0.107
Neurologic Problem	NA	**0.235**	-0.062	-0.083	-0.005	-0.002	-0.025	-0.071	0.076	0.038	0.001	0.093
Headache	NA	**0.15**	0.029	0.128	0.01	-0.077	-0.036	-0.013	**0.141**	-0.114	-0.098	**0.133**
Other Pain	NA	**0.257**	0.085	-0.073	0.079	**-0.267**	**0.133**	-0.009	0.002	-0.121	-0.084	0.022
Recurrent Problem	NA	0.065	0.043	0.124	-0.022	-0.133	**0.157**	0.061	**-0.135**	-0.023	0.025	0.03
Depression	NA	0.042	0.044	-0.104	-0.033	**-0.243**	0.002	0.012	0.018	-0.046	0.066	0.003
Anxiety	NA	0.111	0.02	-0.071	-0.038	-0.073	0.03	0.029	0.01	-0.032	0.072	0.098
Other Psychological Problems	NA	0.095	-0.017	-0.002	0.033	0.018	0.026	0.011	-0.044	0.023	-0.019	**0.131**
Medication for Mental Purposes	NA	0.026	**-0.198**	0.071	-0.155	0.036	-0.169	-0.058	0.045	**0.189**	0.102	0.021

Doctor Visits	NA	0.062	-0.012	-0.106	-0.1	**-0.169**	0.061	0.025	-0.012	-0.01	0.09	-0.099
Other Medication	NA	0.127	0.033	0.133	0.037	-0.082	-0.023	0.071	0.04	-0.082	0.067	0.117
Antibiotic Taken Past 3 Years	NA	0.09	-0.003	-0.044	0.012	0.004	0.006	-0.022	0.082	-0.014	**-0.161**	0.055
Hospitalization Past 5 Years	NA	0.097	-0.015	-0.088	0.073	0.038	0.055	0.009	-0.042	-0.017	0.043	**-0.329**
Life Expectancy	NA	-0.005	0.003	-0.007	0.049	-0.142	0.002	-0.009	0.066	-0.062	-0.097	-0.116
Now Feeling Physically Miserable	NA	**0.14**	-0.019	-0.021	-0.01	-0.023	-0.01	-0.017	-0.01	0.02	-0.026	-0.011
Usually Feeling Physically Miserable	NA	0.004	-0.008	0.045	-0.094	**-0.24**	-0.051	0.095	0.061	-0.092	0.028	0.125
Now Feeling Mentally Miserable	NA	0.019	0.048	0.024	0.028	-0.103	-0.016	0.002	-0.009	-0.047	0.026	0.016
Usually Feeling Mentally Miserable	NA	0.032	0.077	0.044	-0.007	**-0.246**	0.018	**0.167**	-0.006	**-0.138**	0.012	0.098
Year	NA	-0.093	**0.374**	-0.04	**0.436**	**0.207**	**-0.395**	0.107	**0.181**	**0.203**	-0.018	0.004
Course of COVID-19	NA	NA	0.036	**-0.205**	0.105	**0.197**	0.023	-0.045	**0.18**	**-0.145**	**0.136**	-0.051
Months since COVID Infection	NA	0.036	NA	**0.144**	**0.248**	**-0.424**	**0.582**	0.039	**-0.182**	**-0.587**	-0.002	-0.062

Sex (last column) was coded as 1 – men and 2 – women. Significant correlations are bolded. The p-values that remained or turned significant after the application of the Benjamini-Hochberg correction for multiple testing (with FDR set at 0.1) are underlined.

Table S9- P-values of the correlations between health and performance-related variables and COVID-related variables controlled for age, sex, and survey year- All Subjects beyond 24 months post-infection

	Infected	Course	Months since Infection	Vaccination	Months since Vaccination	Covid after Vaccination	Ancestral SARS-CoV-2 variant	Alpha	Delta	Omicron	Age	Sex
Physical Health Issues	NA	0	0.494	0.748	0.923	0.021	0.301	0.459	0.835	0.123	0.816	0.376
Mental Health Issues	NA	0.476	0.663	0.775	0.862	0.067	0.787	0.275	0.984	0.343	0.727	0.284
Fatigue	NA	0.137	0.532	0.106	0.974	0.169	0.065	0.499	0.145	0.808	0.046	0
Intelligence	NA	0.918	0.003	0.009	0.924	0.053	0.027	0.277	0.188	0.007	0.714	0.036
Memory	NA	0.674	0.223	0.049	0.98	0.307	0.095	0.084	0.228	0.322	0.504	0.008
Reactions	NA	0.743	0.164	0.013	0.402	0.34	0.114	0.156	0.326	0.801	0.713	0.258
Accuracy	NA	0.607	0.169	0.032	0.675	0.08	0.128	0.264	0.314	0.094	0.134	0.425
Output Variables												
Evolutionary Biology Score	NA	0.982	0.761	0.709	0.88	0.045	0.416	0.292	0.383	0.604	0.014	0.858
Free-Recall Memory	NA	0.675	0.301	0.278	0.66	0.147	0.196	0.33	0.649	0.495	0.686	0.006
Recognition Memory	NA	0.686	0.265	0.147	0.75	0.132	0.253	0.026	0.274	0.408	0.537	0.003
Simple RT Duration	NA	0.586	0.093	0.009	0.078	0.965	0.138	0.289	0.068	0.842	0.909	0.863
Simple RT Precision	NA	0.732	0.874	0.239	0.48	0.01	0.095	0.389	0.168	0.792	0.852	0.166
Stroop Duration	NA	0.529	0.449	0.089	0.962	0.403	0.652	0.89	0.945	0.628	0.871	0.365
Stroop Precision	NA	0.293	0.643	0.053	0.883	0.621	0.012	0	0.162	0.439	0.374	0.146
Reading Time	NA	0.843	0.186	0.372	0.203	0.74	0.175	0.238	0.831	0.223	0.49	0.159
Allergy	NA	0.006	0.891	0.015	0.88	0.301	0.398	0.007	0.412	0.228	0.803	0.568
Skin Problem	NA	0.002	0.567	0.906	0.145	0.852	0.952	0.841	0.539	0.491	0.848	0.298
Digestive Problem	NA	0.136	0.389	0.488	0.647	0.301	0.827	0.918	0.724	0.252	0.098	0.791
Infection	NA	0.036	0.881	0.038	0.959	0.081	0.314	0.205	0.658	0.915	0.401	0.149
Cardiovascular Problem	NA	0.986	0.646	0.089	0.045	0.064	0.776	0.399	0.644	0.619	0.259	0.452
Low Blood Pressure	NA	0.485	0.911	0.012	0.824	0.136	0.66	0.516	0.806	0.901	0.57	0
High Blood Pressure	NA	0.271	0.267	0.259	0.935	0.158	0.017	0.326	0.104	0.454	0.027	0.359
Orthopedic Problem	NA	0.885	0.002	0.128	0.15	0.062	0.007	0.026	0.393	0	0.354	0.62
Metabolic Problem	NA	0.111	0.065	0.144	0.605	0.496	0.073	0.097	0.007	0.771	0.719	0.1
Neurologic Problem	NA	0	0.345	0.211	0.959	0.987	0.702	0.275	0.245	0.561	0.984	0.156
Headache	NA	0.022	0.659	0.053	0.918	0.414	0.587	0.839	0.031	0.081	0.134	0.042
Other Pain	NA	0	0.194	0.272	0.402	0.005	0.043	0.888	0.973	0.065	0.196	0.733
Recurrent Problem	NA	0.324	0.507	0.061	0.817	0.161	0.017	0.35	0.039	0.721	0.703	0.643
Depression	NA	0.518	0.5	0.116	0.727	0.01	0.97	0.86	0.779	0.476	0.308	0.964
Anxiety	NA	0.089	0.753	0.278	0.69	0.439	0.642	0.658	0.877	0.625	0.266	0.131
Other Psychological Problems	NA	0.148	0.796	0.972	0.729	0.85	0.686	0.869	0.503	0.725	0.773	0.044
Medication for Mental Purposes	NA	0.712	0.004	0.308	0.115	0.715	0.015	0.399	0.517	0.006	0.137	0.756

Doctor Visits	NA	0.346	0.855	0.109	0.292	0.075	0.352	0.706	0.856	0.873	0.166	0.128
Other Medication	NA	0.067	0.629	0.057	0.708	0.401	0.736	0.3	0.558	0.232	0.33	0.089
Antibiotic Taken Past 3 Years	NA	0.168	0.964	0.499	0.903	0.966	0.928	0.737	0.206	0.825	0.013	0.399
Hospitalization Past 5 Years	NA	0.137	0.82	0.181	0.443	0.687	0.398	0.888	0.519	0.799	0.511	0
Life Expectancy	NA	0.941	0.96	0.913	0.604	0.133	0.977	0.896	0.315	0.338	0.135	0.074
Now Feeling Physically Miserable	NA	0.033	0.772	0.754	0.918	0.812	0.877	0.8	0.878	0.76	0.683	0.87
Usually Feeling Physically Miserable	NA	0.947	0.9	0.498	0.321	0.011	0.431	0.144	0.351	0.157	0.662	0.055
Now Feeling Mentally Miserable	NA	0.774	0.464	0.716	0.767	0.277	0.81	0.977	0.889	0.475	0.689	0.805
Usually Feeling Mentally Miserable	NA	0.626	0.235	0.508	0.945	0.009	0.786	0.01	0.93	0.034	0.855	0.131
Year	NA	0.153	0	0.537	0	0.027	0	0.098	0.005	0.002	0.78	0.952
Course of COVID-19	NA	NA	0.586	0.002	0.269	0.038	0.721	0.491	0.006	0.027	0.037	0.43
Months since COVID Infection	NA	0.586	NA	0.028	0.009	0	0	0.551	0.005	0	0.974	0.341

Table S10- Correlations between health and performance-related variables and COVID-related variables controlled for age, sex, and survey year-Women beyond 24 months post-infection

	Infected	Course	Months since Infection	Vaccination	Months since Vaccination	Covid after Vaccination	Ancestral SARS-CoV-2 variant	Alpha	Delta	Omicron	Age	Sex
Physical Health Issues	NA	**0.194**	0.052	-0.004	-0.039	-0.189	0.111	-0.038	0.027	-0.093	-0.001	NA
Mental Health Issues	NA	0.059	0.036	0.025	0.036	-0.204	-0.002	-0.003	-0.052	0.004	-0.04	NA
Fatigue	NA	0.118	0.08	0.099	0.105	-0.064	**0.175**	-0.079	-0.115	-0.035	0.12	NA
Intelligence	NA	-0.033	**-0.184**	-0.136	-0.054	0.199	-0.125	-0.074	0.094	**0.159**	-0.045	NA
Memory	NA	-0.006	-0.021	**-0.149**	0.017	0.056	-0.055	-0.068	0.034	0.001	-0.034	NA
Reactions	NA	0.038	0.073	**-0.197**	0.081	-0.065	0.03	-0.048	-0.036	0.003	-0.006	NA
Accuracy	NA	-0.008	-0.08	**-0.15**	-0.036	**0.261**	-0.091	-0.132	0.141	0.069	-0.067	NA
Output Variables												
Evolutionary Biology Score	NA	0.037	0.046	-0.012	-0.002	**0.268**	-0.03	0.047	0.104	-0.085	-0.11	NA
Free-Recall Memory	NA	-0.004	-0.008	-0.09	0.029	0.104	-0.014	-0.043	-0.022	-0.017	-0.006	NA
Recognition Memory	NA	0	-0.014	-0.109	0.014	0.109	-0.021	-0.108	0.028	-0.008	-0.04	NA
Simple RT Duration	NA	-0.019	0.092	**-0.207**	0.166	0.061	0.045	-0.09	-0.067	0.024	-0.015	NA
Simple RT Precision	NA	0.023	-0.011	-0.088	0.096	**-0.28**	-0.127	0.065	0.105	-0.019	0.012	NA
Stroop Duration	NA	0.061	0.029	-0.14	0.014	-0.07	-0.018	0.004	0.015	-0.006	-0.029	NA
Stroop Precision	NA	-0.04	0.028	-0.138	0.022	0.198	**0.156**	**-0.32**	-0.036	0.046	-0.063	NA
Reading Time	NA	-0.055	0.091	-0.061	0.145	0.066	0.069	-0.013	-0.059	-0.066	0.037	NA
Allergy	NA	0.145	0.019	**0.178**	0.073	-0.029	0.113	0.105	-0.113	-0.024	-0.037	NA
Skin Problem	NA	**0.226**	0.031	0.008	-0.08	0.086	-0.007	-0.038	0.098	-0.05	0.08	NA
Digestive Problem	NA	0.089	-0.049	-0.082	0.036	-0.074	0.048	-0.033	-0.049	0.055	**0.168**	NA
Infection	NA	0.129	0.006	**-0.165**	-0.02	**0.234**	0.055	**-0.193**	**0.156**	-0.01	-0.049	NA
Cardiovascular Problem	NA	-0.015	-0.045	0.107	**-0.246**	-0.202	0.013	-0.078	-0.002	0.059	-0.036	NA
Low Blood Pressure	NA	-0.095	0.019	**-0.227**	-0.016	**-0.243**	-0.029	0.11	-0.098	0.036	-0.009	NA
High Blood Pressure	NA	0.199	-0.061	-0.237	-0.324	0.055	-0.226	0.202	0.226	-0.048	**0.257**	NA
Orthopedic Problem	NA	0.01	**0.254**	0.132	0.192	-0.21	**0.235**	**0.17**	-0.074	**-0.313**	0.036	NA
Metabolic Problem	NA	0.029	-0.106	-0.072	-0.068	0.071	-0.121	-0.109	**0.203**	-0.004	0.032	NA
Neurologic Problem	NA	**0.229**	-0.049	-0.118	-0.076	-0.006	0.009	-0.079	0.04	0.05	0.004	NA
Headache	NA	0.13	0.056	0.133	0.01	-0.099	0.061	-0.076	0.055	-0.1	**-0.151**	NA
Other Pain	NA	**0.249**	0.102	-0.071	0.106	**-0.221**	**0.202**	-0.091	0.002	-0.133	-0.132	NA
Recurrent Problem	NA	0.085	-0.021	0.093	-0.018	-0.106	0.141	-0.016	-0.116	0.042	0.005	NA
Depression	NA	0.085	0.072	-0.077	-0.018	**-0.235**	0.033	-0.087	0.038	-0.037	0.076	NA
Anxiety	NA	0.139	0.035	-0.094	0.002	-0.05	0.096	-0.022	-0.1	0.041	0.043	NA
Other Psychological Problems	NA	0.134	-0.017	-0.02	0.084	0.06	0.032	-0.044	-0.047	0.06	-0.123	NA
Medication for Mental Purposes	NA	-0.017	**-0.227**	0.101	**-0.257**	0.054	**-0.2**	-0.074	0.063	**0.215**	0.092	NA
Doctor Visits	NA	0.016	-0.059	-0.094	-0.182	-0.136	0.044	-0.069	0.073	0.009	0.082	NA
Other Medication	NA	0.11	0.032	0.134	0.048	-0.098	-0.029	-0.024	0.096	-0.071	0.086	NA

Antibiotic Taken Past 3 Years	NA	0.022	-0.026	0.01	-0.072	-0.023	-0.014	-0.045	0.125	0	-0.127	NA
Hospitalization Past 5 Years	NA	-0.064	0.036	0.093	0.011	-0.089	**0.186**	-0.073	-0.12	-0.024	0.037	NA
Life Expectancy	NA	-0.022	0.018	0.073	0.074	-0.173	0.003	-0.017	0.132	-0.116	-0.061	NA
Now Feeling Physically Miserable	NA	0.135	-0.028	-0.029	-0.085	0.014	-0.014	-0.077	-0.005	0.056	-0.078	NA
Usually Feeling Physically Miserable	NA	-0.024	-0.006	0.042	-0.123	-0.153	-0.053	0.092	0.046	-0.079	0.048	NA
Now Feeling Mentally Miserable	NA	-0.028	0.08	0.061	0.02	**-0.241**	0.024	-0.092	-0.074	0.009	-0.064	NA
Usually Feeling Mentally Miserable	NA	0.065	0.077	0.008	0.031	-0.18	0.027	0.098	-0.039	-0.073	-0.028	NA
Year	NA	-0.06	**0.34**	-0.017	**0.449**	0.207	**-0.407**	0.106	**0.171**	**0.218**	-0.051	NA
Course of COVID-19	NA	NA	0.045	**-0.184**	0.067	**0.335**	0.059	-0.08	**0.164**	-0.131	0.116	NA
Months since COVID Infection	NA	0.045	NA	0.12	**0.283**	**-0.417**	**0.564**	0.049	**-0.153**	**-0.604**	-0.026	NA

Significant correlations are bolded. The p-values that remained or turned significant after the application of the Benjamini-Hochberg correction for multiple testing (with FDR set at 0.1) are underlined.

Table S11- P-values of the correlations between health and performance-related variables and COVID-related variables controlled for age, sex, and survey year- Women beyond 24 months post-infection

	Infected	Course	Months since Infection	Vaccination	Months since Vaccination	Covid after Vaccination	Ancestral SARS-CoV-2 variant	Alpha	Delta	Omicron	Age	Sex
Physical Health Issues	NA	0.01	0.483	0.962	0.722	0.086	0.135	0.608	0.714	0.211	0.985	NA
Mental Health Issues	NA	0.428	0.625	0.738	0.74	0.064	0.979	0.971	0.481	0.955	0.588	NA
Fatigue	NA	0.114	0.281	0.19	0.341	0.563	0.018	0.287	0.122	0.64	0.104	NA
Intelligence	NA	0.663	0.013	0.071	0.622	0.071	0.093	0.322	0.205	0.033	0.54	NA
Memory	NA	0.932	0.78	0.048	0.877	0.611	0.459	0.356	0.649	0.995	0.649	NA
Reactions	NA	0.607	0.329	0.009	0.46	0.557	0.689	0.521	0.625	0.969	0.937	NA
Accuracy	NA	0.92	0.282	0.046	0.745	0.018	0.22	0.076	0.057	0.353	0.362	NA
Output Variables												
Evolutionary Biology Score	NA	0.622	0.536	0.878	0.989	0.015	0.688	0.531	0.162	0.253	0.139	NA
Free-Recall Memory	NA	0.96	0.918	0.256	0.794	0.352	0.854	0.585	0.779	0.831	0.934	NA
Recognition Memory	NA	1	0.86	0.157	0.898	0.32	0.784	0.16	0.715	0.917	0.602	NA
Simple RT Duration	NA	0.796	0.213	0.006	0.131	0.578	0.543	0.225	0.37	0.749	0.836	NA
Simple RT Precision	NA	0.761	0.877	0.242	0.385	0.011	0.088	0.382	0.157	0.799	0.875	NA
Stroop Duration	NA	0.411	0.696	0.062	0.898	0.524	0.806	0.953	0.839	0.933	0.699	NA
Stroop Precision	NA	0.59	0.712	0.069	0.838	0.072	0.037	0	0.627	0.539	0.398	NA
Reading Time	NA	0.465	0.222	0.418	0.189	0.55	0.354	0.86	0.426	0.375	0.615	NA
Allergy	NA	0.053	0.797	0.018	0.509	0.794	0.129	0.158	0.127	0.751	0.611	NA
Skin Problem	NA	0.003	0.674	0.914	0.469	0.433	0.93	0.611	0.189	0.504	0.278	NA
Digestive Problem	NA	0.235	0.51	0.273	0.74	0.5	0.52	0.658	0.509	0.462	0.023	NA
Infection	NA	0.084	0.941	0.028	0.858	0.033	0.456	0.009	0.035	0.895	0.509	NA
Cardiovascular Problem	NA	0.844	0.542	0.154	0.025	0.067	0.856	0.294	0.982	0.429	0.628	NA
Low Blood Pressure	NA	0.249	0.816	0.006	0.89	0.04	0.721	0.177	0.231	0.656	0.915	NA
High Blood Pressure	NA	0.103	0.614	0.057	0.08	0.765	0.06	0.093	0.06	0.688	0.03	NA
Orthopedic Problem	NA	0.894	0.001	0.08	0.081	0.056	0.002	0.022	0.318	0	0.623	NA
Metabolic Problem	NA	0.697	0.152	0.337	0.536	0.517	0.104	0.143	0.006	0.959	0.666	NA
Neurologic Problem	NA	0.002	0.508	0.115	0.488	0.957	0.9	0.289	0.589	0.505	0.959	NA
Headache	NA	0.082	0.449	0.076	0.929	0.367	0.413	0.303	0.463	0.177	0.041	NA
Other Pain	NA	0.001	0.17	0.344	0.335	0.045	0.006	0.221	0.976	0.073	0.073	NA
Recurrent Problem	NA	0.257	0.773	0.215	0.872	0.335	0.058	0.831	0.119	0.569	0.951	NA
Depression	NA	0.258	0.333	0.308	0.872	0.033	0.659	0.242	0.611	0.615	0.301	NA
Anxiety	NA	0.064	0.638	0.209	0.983	0.652	0.197	0.77	0.179	0.582	0.564	NA
Other Psychological Problems	NA	0.074	0.824	0.791	0.443	0.587	0.662	0.557	0.531	0.422	0.095	NA
Medication for Mental Purposes	NA	0.834	0.004	0.211	0.025	0.637	0.012	0.35	0.427	0.007	0.242	NA
Doctor Visits	NA	0.83	0.427	0.211	0.098	0.218	0.556	0.353	0.328	0.899	0.269	NA
Other Medication	NA	0.165	0.681	0.093	0.672	0.385	0.716	0.757	0.224	0.368	0.27	NA

Antibiotic Taken Past 3 Years	NA	0.773	0.722	0.89	0.512	0.834	0.851	0.544	0.092	1	0.085	NA
Hospitalization Past 5 Years	NA	0.391	0.626	0.218	0.923	0.419	0.012	0.323	0.107	0.747	0.619	NA
Life Expectancy	NA	0.763	0.809	0.335	0.504	0.115	0.972	0.817	0.075	0.119	0.408	NA
Now Feeling Physically Miserable	NA	0.07	0.71	0.703	0.441	0.9	0.848	0.3	0.951	0.449	0.29	NA
Usually Feeling Physically Miserable	NA	0.749	0.933	0.58	0.262	0.164	0.477	0.217	0.532	0.286	0.517	NA
Now Feeling Mentally Miserable	NA	0.711	0.28	0.416	0.859	0.028	0.747	0.216	0.317	0.9	0.383	NA
Usually Feeling Mentally Miserable	NA	0.388	0.301	0.912	0.781	0.103	0.717	0.185	0.597	0.324	0.707	NA
Year	NA	0.42	0	0.822	0	0.057	0	0.153	0.021	0.003	0.485	NA
Course of COVID-19	NA	NA	0.549	0.014	0.543	0.002	0.43	0.283	0.029	0.079	0.118	NA
Months since COVID Infection	NA	0.549	NA	0.111	0.01	0	0	0.509	0.039	0	0.725	NA

Table S12- Correlations between health and performance-related variables and COVID-related variables controlled for age, sex, and survey year- Men beyond 24 months post-infection

	Infected	Course	Months since Infection	Vaccination	Months since Vaccination	Covid after Vaccination	Ancestral SARS-CoV-2 variant	Alpha	Delta	Omicron	Age	Sex
Physical Health Issues	NA	**0.367**	0.031	-0.105	-0.06	-0.319	-0.072	0.289	-0.006	-0.12	0.06	NA
Mental Health Issues	NA	0.021	-0.017	0.025	-0.192	-0.034	-0.118	0.291	0.176	-0.274	0.204	NA
Fatigue	NA	-0.11	-0.069	0.127	**-0.538**	-0.373	-0.036	0.022	-0.009	0.039	0.191	NA
Intelligence	NA	0.147	-0.191	**-0.401**	0.103	0.157	-0.236	-0.076	0.095	0.242	0.092	NA
Memory	NA	0.041	-0.264	-0.051	0.023	0.205	-0.269	**-0.333**	0.264	0.283	-0.039	NA
Reactions	NA	-0.074	0.209	-0.022	0.214	-0.152	**0.369**	**-0.313**	-0.133	-0.073	0.207	NA
Accuracy	NA	-0.21	-0.081	-0.136	-0.113	-0.282	-0.102	0.191	-0.238	0.259	-0.216	NA
Output Variables												
Evolutionary Biology Score	NA	-0.124	-0.04	0.22	-0.032	-0.157	-0.106	0.168	-0.142	0.171	**-0.399**	NA
Free-Recall Memory	NA	0.074	-0.259	-0.049	0.077	0.215	**-0.334**	-0.19	0.199	0.293	-0.04	NA
Recognition Memory	NA	0.035	-0.264	-0.083	0.077	0.215	-0.278	**-0.334**	0.242	**0.319**	0.055	NA
Simple RT Duration	NA	-0.145	0.253	0.026	0.236	-0.133	**0.301**	0.032	**-0.317**	-0.035	0.138	NA
Simple RT Precision	NA	NA	NA	NA	NA	NA	NA	NA	NA	NA	NA	NA
Stroop Duration	NA	-0.043	0.24	0.011	0.023	-0.175	0.251	-0.055	-0.07	-0.193	0.11	NA
Stroop Precision	NA	-0.267	0.046	-0.063	-0.237	**-0.522**	0.221	0.067	**-0.367**	0.097	-0.045	NA
Reading Time	NA	0.156	0.154	-0.083	0.014	-0.13	0.196	**-0.323**	0.149	-0.174	0.138	NA
Allergy	NA	**0.307**	-0.083	0.084	**-0.442**	-0.319	-0.168	**0.394**	0.152	-0.261	0.209	NA
Skin Problem	NA	0.144	0.114	-0.092	-0.351	-0.315	0.116	0.183	-0.155	-0.103	-0.241	NA
Digestive Problem	NA	0.133	-0.085	0.085	**-0.451**	-0.215	-0.194	0.09	0.044	0.125	-0.16	NA
Infection	NA	0.126	0.142	-0.033	-0.17	-0.109	0.135	**0.331**	**-0.408**	0.053	-0.049	NA
Cardiovascular Problem	NA	0.096	0.045	0.11	NA	NA	-0.138	**0.7**	-0.179	-0.147	-0.254	NA
Low Blood Pressure	NA	0.183	-0.08	0.087	0.18	0.197	-0.092	-0.158	0.327	-0.158	0.277	NA
High Blood Pressure	NA	-0.242	-0.188	0.048	0.45	0.507	-0.242	-0.08	-0.086	**0.447**	0.132	NA
Orthopedic Problem	NA	0	-0.046	-0.08	-0.156	-0.08	-0.073	0.036	0.026	0.036	0.173	NA
Metabolic Problem	NA	**0.439**	-0.172	-0.26	-0.146	0.026	-0.092	-0.11	0.076	0.111	0.02	NA
Neurologic Problem	NA	0.277	-0.103	0.045	0.103	-0.008	-0.192	-0.051	**0.32**	-0.073	0.074	NA
Headache	NA	0.246	-0.196	0.068	-0.115	0.018	**-0.462**	0.189	**0.489**	-0.103	0.022	NA
Other Pain	NA	0.239	-0.036	-0.085	-0.115	**-0.445**	-0.11	0.225	-0.002	-0.031	0.037	NA
Recurrent Problem	NA	-0.016	0.299	0.28	-0.044	-0.229	0.225	**0.34**	-0.229	-0.286	0.081	NA
Depression	NA	-0.118	-0.04	-0.225	-0.174	-0.264	-0.079	**0.329**	-0.048	-0.093	0.037	NA
Anxiety	NA	-0.023	-0.08	0.057	-0.357	-0.176	-0.208	0.209	**0.345**	**-0.295**	0.141	NA
Other Psychological Problems	NA	-0.1	-0.121	0.156	-0.176	-0.107	-0.099	0.197	0.016	-0.043	**0.463**	NA
Medication for Mental Purposes	NA	0.134	-0.029	-0.035	0.12	-0.028	-0.079	0.015	0.043	0.038	0.282	NA
Doctor Visits	NA	0.265	0.2	-0.16	0.176	-0.328	0.129	**0.396**	**-0.313**	-0.099	0.116	NA
Other Medication	NA	0.225	0.045	0.112	NA	NA	0.034	**0.445**	-0.194	-0.168	0.001	NA

Antibiotic Taken Past 3 Years	NA	**0.373**	0.16	**-0.338**	0.368	0.119	0.126	0.074	-0.075	-0.126	**-0.298**	NA
Hospitalization Past 5 Years	NA	**0.372**	-0.076	**-0.455**	0.149	0.17	-0.094	0.106	0.03	0.004	0.076	NA
Life Expectancy	NA	0.062	0.013	**-0.486**	-0.099	-0.032	0.067	0.016	-0.178	0.109	-0.232	NA
Now Feeling Physically Miserable	NA	0.15	-0.012	0.005	0.285	-0.201	-0.041	0.19	-0.013	-0.077	0.2	NA
Usually Feeling Physically Miserable	NA	0.103	-0.015	0.033	-0.166	**-0.555**	-0.019	0.116	0.109	-0.189	-0.033	NA
Now Feeling Mentally Miserable	NA	0.126	-0.109	-0.083	-0.05	0.265	-0.211	**0.305**	0.216	-0.215	**0.365**	NA
Usually Feeling Mentally Miserable	NA	-0.055	0.064	0.202	-0.323	**-0.524**	-0.018	**0.339**	0.103	**-0.351**	0.103	NA
Year	NA	-0.205	**0.462**	-0.123	0.381	0.209	**-0.388**	0.114	0.222	0.176	0.093	NA
Course of COVID-19	NA	NA	0.015	**-0.325**	0.162	-0.195	-0.12	0.074	0.248	-0.193	0.19	NA
Months since COVID Infection	NA	0.015	NA	0.279	0.142	-0.426	**0.67**	0.007	-0.288	**-0.512**	0.116	NA

Significant correlations are bolded. The p-values that remained or turned significant after the application of the Benjamini-Hochberg correction for multiple testing (with FDR set at 0.1) are underlined.

Table S13- P-values of the correlations between health and performance-related variables and COVID-related variables controlled for age, sex, and survey year- Men beyond 24 months post-infection

	Infected	Course	Months since Infection	Vaccination	Months since Vaccination	Covid after Vaccination	Ancestral SARS-CoV-2 variant	Alpha	Delta	Omicron	Age	Sex
Physical Health Issues	NA	0.014	0.835	0.483	0.787	0.149	0.63	0.054	0.966	0.424	0.683	NA
Mental Health Issues	NA	0.889	0.911	0.869	0.385	0.877	0.429	0.052	0.238	0.067	0.162	NA
Fatigue	NA	0.463	0.647	0.395	0.015	0.092	0.812	0.881	0.95	0.797	0.19	NA
Intelligence	NA	0.325	0.201	0.007	0.642	0.477	0.115	0.612	0.524	0.105	0.529	NA
Memory	NA	0.784	0.078	0.734	0.916	0.353	0.072	0.026	0.078	0.058	0.792	NA
Reactions	NA	0.619	0.162	0.882	0.332	0.493	0.014	0.037	0.374	0.626	0.157	NA
Accuracy	NA	0.16	0.59	0.363	0.608	0.201	0.496	0.202	0.112	0.084	0.139	NA
Output Variables												
Evolutionary Biology Score	NA	0.406	0.79	0.142	0.884	0.476	0.478	0.262	0.342	0.254	0.006	NA
Free-Recall Memory	NA	0.638	0.101	0.757	0.742	0.358	0.034	0.228	0.206	0.063	0.795	NA
Recognition Memory	NA	0.822	0.094	0.599	0.742	0.358	0.078	0.034	0.124	0.043	0.718	NA
Simple RT Duration	NA	0.331	0.091	0.861	0.286	0.548	0.044	0.833	0.034	0.816	0.346	NA
Simple RT Precision	NA	NA	NA	NA	NA	NA	NA	NA	NA	NA	NA	NA
Stroop Duration	NA	0.772	0.109	0.941	0.917	0.429	0.093	0.713	0.642	0.197	0.453	NA
Stroop Precision	NA	0.074	0.76	0.672	0.284	0.018	0.14	0.656	0.014	0.515	0.758	NA
Reading Time	NA	0.297	0.304	0.58	0.948	0.555	0.191	0.031	0.321	0.245	0.345	NA
Allergy	NA	0.04	0.578	0.574	0.045	0.149	0.262	0.009	0.311	0.081	0.153	NA
Skin Problem	NA	0.337	0.448	0.537	0.112	0.154	0.439	0.221	0.299	0.49	0.099	NA
Digestive Problem	NA	0.373	0.569	0.57	0.041	0.329	0.194	0.549	0.768	0.404	0.274	NA
Infection	NA	0.398	0.344	0.824	0.443	0.621	0.367	0.027	0.006	0.724	0.735	NA
Cardiovascular Problem	NA	0.522	0.765	0.463	NA	NA	0.357	0	0.232	0.326	0.082	NA
Low Blood Pressure	NA	0.288	0.641	0.614	0.5	0.459	0.593	0.359	0.058	0.359	0.097	NA
High Blood Pressure	NA	0.209	0.33	0.802	0.119	0.079	0.208	0.677	0.656	0.02	0.475	NA
Orthopedic Problem	NA	1	0.757	0.591	0.479	0.718	0.624	0.812	0.864	0.812	0.237	NA
Metabolic Problem	NA	0.003	0.251	0.083	0.509	0.907	0.539	0.461	0.613	0.459	0.889	NA
Neurologic Problem	NA	0.071	0.503	0.769	0.64	0.973	0.212	0.738	0.037	0.636	0.622	NA
Headache	NA	0.109	0.203	0.658	0.602	0.936	0.003	0.218	0.001	0.501	0.885	NA
Other Pain	NA	0.12	0.814	0.581	0.603	0.044	0.473	0.143	0.99	0.84	0.806	NA
Recurrent Problem	NA	0.915	0.051	0.069	0.842	0.299	0.143	0.027	0.135	0.063	0.59	NA
Depression	NA	0.432	0.792	0.133	0.431	0.233	0.597	0.028	0.749	0.535	0.798	NA
Anxiety	NA	0.877	0.592	0.706	0.106	0.426	0.165	0.163	0.021	0.049	0.333	NA
Other Psychological Problems	NA	0.515	0.429	0.311	0.426	0.627	0.517	0.2	0.919	0.779	0.002	NA
Medication for Mental Purposes	NA	0.396	0.852	0.826	0.607	0.903	0.618	0.922	0.787	0.811	0.066	NA
Doctor Visits	NA	0.077	0.181	0.285	0.426	0.138	0.388	0.008	0.037	0.51	0.428	NA
Other Medication	NA	0.153	0.776	0.479	NA	NA	0.827	0.005	0.218	0.287	0.994	NA

Antibiotic Taken Past 3 Years	NA	0.013	0.286	0.024	0.096	0.591	0.401	0.623	0.615	0.401	0.041	NA
Hospitalization Past 5 Years	NA	0.013	0.613	0.002	0.501	0.442	0.529	0.479	0.841	0.98	0.603	NA
Life Expectancy	NA	0.679	0.929	0.001	0.656	0.883	0.653	0.913	0.234	0.466	0.112	NA
Now Feeling Physically Miserable	NA	0.315	0.938	0.971	0.197	0.362	0.784	0.204	0.931	0.607	0.171	NA
Usually Feeling Physically Miserable	NA	0.493	0.922	0.823	0.453	0.012	0.897	0.44	0.465	0.206	0.82	NA
Now Feeling Mentally Miserable	NA	0.399	0.467	0.578	0.822	0.23	0.159	0.041	0.149	0.151	0.012	NA
Usually Feeling Mentally Miserable	NA	0.712	0.667	0.178	0.143	0.018	0.906	0.024	0.493	0.019	0.48	NA
Year	NA	0.161	0.002	0.401	0.07	0.32	0.008	0.436	0.129	0.229	0.513	NA
Course of COVID-19	NA	NA	0.922	0.03	0.463	0.378	0.424	0.62	0.097	0.198	0.193	NA
Months since COVID Infection	NA	0.922	NA	0.062	0.52	0.054	0	0.964	0.054	0.001	0.426	NA

Significant correlations are bolded. The p-values that remained or turned significant after the application of the Benjamini-Hochberg correction for multiple testing (with FDR set at 0.1) are underlined.

Table S14- Correlations between health and performance-related variables and COVID-related variables controlled for age, sex, and survey year- all subjects with at least 36 months elapsed since infection

	Infected	Course	Months since Infection	Vaccination	Months since Vaccination	Covid after Vaccination	Ancestral SARS-CoV-2 variant	Alpha	Delta	Omicron	Age	Sex
Physical Health Issues	NA	**0.329**	-0.028	-0.081	0.088	NA	0.041	0.016	-0.045	NA	-0.101	0
Mental Health Issues	NA	0.088	0.037	-0.086	0.019	NA	-0.126	0.086	-0.061	NA	-0.033	0.039
Fatigue	NA	0.073	0.009	0.192	-0.184	NA	0.144	-0.148	0.017	NA	0.085	**0.393**
Intelligence	NA	-0.041	-0.072	-0.192	0.024	NA	0.106	-0.013	-0.012	NA	-0.038	-0.157
Memory	NA	-0.125	0.038	**-0.218**	-0.031	NA	-0.055	-0.136	0.168	NA	-0.022	**0.318**
Reactions	NA	0.093	0.093	**-0.233**	0.011	NA	0.108	-0.18	0.035	NA	-0.027	0.038
Accuracy	NA	0.04	0.125	**-0.217**	0.027	NA	0.085	-0.035	-0.142	NA	-0.162	-0.153
Evolutionary Biology Score	NA	0.098	0.015	-0.023	0.077	NA	-0.045	0.107	-0.128	NA	**-0.288**	-0.03
Free-Recall Memory	NA	-0.098	-0.025	-0.179	0.007	NA	-0.107	-0.12	0.2	NA	-0.009	**0.423**
Recognition Memory	NA	-0.111	0.032	-0.184	-0.018	NA	0	**-0.217**	0.193	NA	0.009	**0.421**
Simple RT Duration	NA	0.143	**0.235**	**-0.246**	0.211	NA	0.11	-0.141	-0.17	NA	0.005	-0.06
Simple RT Precision	NA	-0.06	0.084	-0.068	NA	NA	-0.134	0.097	0.038	NA	0.009	-0.107
Stroop Duration	NA	-0.012	-0.053	-0.003	-0.005	NA	-0.019	-0.044	0.14	NA	-0.087	-0.01
Stroop Precision	NA	0.039	**0.341**	-0.112	0.066	NA	**0.391**	**-0.369**	**-0.389**	NA	-0.014	-0.184
Reading Time	NA	-0.044	0.104	-0.033	0.051	NA	0.041	-0.134	0.033	NA	0.072	0.186
Allergy	NA	**0.216**	**-0.251**	0.02	-0.122	NA	-0.089	**0.205**	0.062	NA	-0.045	0.035
Skin Problem	NA	0.17	0.013	-0.107	-0.065	NA	-0.015	-0.002	0.104	NA	-0.155	-0.159
Digestive Problem	NA	0.041	-0.03	-0.004	0.125	NA	0.068	0.011	-0.018	NA	0.099	0.055
Infection	NA	**0.197**	0.085	-0.187	-0.176	NA	0.155	-0.107	0.105	NA	-0.022	-0.013
Cardiovascular Problem	NA	-0.001	0.032	0.067	-0.262	NA	-0.031	0.089	-0.037	NA	-0.171	-0.092
Low Blood Pressure	NA	-0.126	0.014	-0.196	0.281	NA	0.004	0.064	-0.21	NA	-0.2	**0.329**
High Blood Pressure	NA	-0.149	-0.018	NA	-0.2	NA	-0.164	**0.304**	-0.113	NA	-0.043	0.234
Orthopedic Problem	NA	-0.004	0.016	0	0.045	NA	0.046	0.052	**-0.203**	NA	0.037	0.171
Metabolic Problem	NA	**0.267**	-0.01	-0.071	0.289	NA	0.032	-0.081	-0.071	NA	0.122	0.151
Neurologic Problem	NA	**0.224**	0.098	-0.099	-0.056	NA	0.177	-0.08	-0.083	NA	-0.068	**0.216**
Headache	NA	0.147	0.092	0.093	0.016	NA	-0.047	-0.04	0.063	NA	**-0.22**	**0.262**
Other Pain	NA	0.11	0.03	**-0.228**	0.116	NA	0.167	-0.089	-0.066	NA	**-0.255**	0.006
Recurrent Problem	NA	0.016	-0.05	0.058	-0.045	NA	0.108	0.045	-0.009	NA	-0.115	-0.124
Depression	NA	0.086	0.13	0.026	-0.01	NA	-0.045	0.003	0.013	NA	0.024	-0.032
Anxiety	NA	0.07	0.044	-0.073	-0.049	NA	0.061	0.035	-0.039	NA	-0.018	0.131
Other Psychological Problems	NA	0.193	-0.075	-0.124	0.101	NA	0.02	-0.001	0.058	NA	-0.053	0.067

Medication for Mental Purposes	NA	NA	NA	NA	NA	NA	NA	NA	NA	NA	NA	NA
Doctor Visits	NA	0.157	-0.107	-0.123	-0.221	NA	0.054	0.008	**0.217**	NA	0.006	**-0.252**
Other Medication	NA	0.198	-0.04	0.135	0.098	NA	-0.108	0.082	-0.075	NA	-0.025	0.005
Antibiotic Taken Past 3 Years	NA	0.151	-0.034	**0.196**	-0.073	NA	0.055	-0.021	**0.211**	NA	-0.185	-0.008
Hospitalization Past 5 Years	NA	0.032	-0.119	0.063	-0.148	NA	0.061	-0.019	-0.035	NA	-0.062	**-0.248**
Life Expectancy	NA	0.036	-0.001	0.007	0.022	NA	-0.006	-0.043	0.14	NA	**-0.261**	-0.128
Now Feeling Physically Miserable	NA	**0.214**	0.059	-0.192	0.102	NA	0.027	-0.021	-0.123	NA	-0.12	-0.077
Usually Feeling Physically Miserable	NA	0.081	-0.065	0.022	0.059	NA	-0.135	0.124	-0.076	NA	-0.046	0.144
Now Feeling Mentally Miserable	NA	0.087	0.105	-0.156	0.076	NA	-0.093	-0.025	-0.08	NA	0.044	0.007
Usually Feeling Mentally Miserable	NA	0.083	-0.012	-0.024	-0.041	NA	-0.158	**0.194**	-0.086	NA	-0.031	0.022
Year	NA	NA	NA	NA	NA	NA	NA	NA	NA	NA	NA	NA
Course of COVID-19	NA	NA	0.036	-0.088	0.041	NA	0.135	-0.055	-0.127	NA	0.112	-0.036
Months since COVID Infection	NA	0.036	NA	-0.019	0.158	NA	**0.342**	**-0.526**	**-0.198**	NA	-0.039	-0.042

Sex (last column) was coded as 1 – men and 2 – women. Significant correlations are bolded. The p-values that remained or turned significant after the application of the Benjamini-Hochberg correction for multiple testing (with FDR set at 0.1) are underlined.

Table S15- P-values of the correlations between health and performance-related variables and COVID-related variables controlled for age, sex, and survey year- all subjects with at least 36 months elapsed since infection

	Infected	Course	Months since Infection	Vaccination	Months since Vaccination	Covid after Vaccination	Ancestral SARS-CoV-2 variant	Alpha	Delta	Omicron	Age	Sex
Physical Health Issues	NA	0.001	0.778	0.413	0.556	NA	0.679	0.873	0.648	NA	0.303	0.998
Mental Health Issues	NA	0.372	0.705	0.384	0.898	NA	0.201	0.384	0.54	NA	0.734	0.689
Fatigue	NA	0.459	0.929	0.052	0.22	NA	0.146	0.132	0.867	NA	0.384	0
Intelligence	NA	0.677	0.467	0.051	0.874	NA	0.283	0.893	0.904	NA	0.693	0.108
Memory	NA	0.206	0.698	0.027	0.836	NA	0.577	0.168	0.088	NA	0.824	0.001
Reactions	NA	0.346	0.348	0.018	0.94	NA	0.273	0.068	0.719	NA	0.785	0.698
Accuracy	NA	0.683	0.205	0.028	0.858	NA	0.39	0.72	0.15	NA	0.097	0.117
Evolutionary Biology Score	NA	0.325	0.878	0.819	0.607	NA	0.654	0.282	0.198	NA	0.003	0.761
Free-Recall Memory	NA	0.352	0.81	0.092	0.962	NA	0.312	0.256	0.058	NA	0.934	0
Recognition Memory	NA	0.277	0.755	0.071	0.905	NA	0.997	0.033	0.058	NA	0.929	0
Simple RT Duration	NA	0.149	0.017	0.013	0.158	NA	0.264	0.154	0.085	NA	0.958	0.539
Simple RT Precision	NA	0.545	0.392	0.494	25	NA	0.175	0.327	0.701	NA	0.925	0.271
Stroop Duration	NA	0.905	0.592	0.978	0.973	NA	0.848	0.657	0.156	NA	0.371	0.92
Stroop Precision	NA	0.696	0.001	0.263	0.661	NA	0	0	0	NA	0.884	0.062
Reading Time	NA	0.659	0.294	0.738	0.735	NA	0.675	0.176	0.736	NA	0.463	0.057
Allergy	NA	0.028	0.011	0.84	0.414	NA	0.369	0.037	0.529	NA	0.646	0.717
Skin Problem	NA	0.084	0.898	0.28	0.664	NA	0.878	0.986	0.29	NA	0.112	0.103
Digestive Problem	NA	0.676	0.763	0.967	0.403	NA	0.493	0.91	0.855	NA	0.31	0.571
Infection	NA	0.046	0.389	0.058	0.24	NA	0.115	0.277	0.288	NA	0.824	0.896
Cardiovascular Problem	NA	0.988	0.749	0.495	0.081	NA	0.751	0.369	0.706	NA	0.08	0.347
Low Blood Pressure	NA	0.241	0.895	0.067	0.083	NA	0.973	0.553	0.05	NA	0.059	0.002
High Blood Pressure	NA	0.331	0.908	2	0.453	NA	0.286	0.047	0.462	NA	0.772	0.118
Orthopedic Problem	NA	0.97	0.869	0.998	0.766	NA	0.641	0.601	0.039	NA	0.701	0.08
Metabolic Problem	NA	0.007	0.915	0.474	0.053	NA	0.748	0.412	0.473	NA	0.212	0.122
Neurologic Problem	NA	0.023	0.318	0.317	0.707	NA	0.073	0.417	0.402	NA	0.489	0.027
Headache	NA	0.136	0.349	0.346	0.916	NA	0.634	0.684	0.523	NA	0.024	0.007
Other Pain	NA	0.266	0.759	0.021	0.44	NA	0.091	0.364	0.505	NA	0.009	0.947
Recurrent Problem	NA	0.875	0.615	0.557	0.762	NA	0.272	0.651	0.924	NA	0.238	0.204
Depression	NA	0.386	0.187	0.791	0.947	NA	0.651	0.976	0.895	NA	0.802	0.742
Anxiety	NA	0.481	0.659	0.46	0.743	NA	0.534	0.723	0.69	NA	0.851	0.179
Other Psychological Problems	NA	0.053	0.455	0.215	0.499	NA	0.84	0.992	0.562	NA	0.589	0.499

Medication for Mental Purposes	NA	NA	NA	NA	NA	NA	NA	NA	NA	NA	NA	NA
Doctor Visits	NA	0.112	0.28	0.213	0.14	NA	0.585	0.933	0.028	NA	0.952	0.01
Other Medication	NA	0.062	0.705	0.201	0.525	NA	0.308	0.439	0.476	NA	0.808	0.963
Antibiotic Taken Past 3 Years	NA	0.126	0.727	0.047	0.628	NA	0.577	0.832	0.033	NA	0.058	0.932
Hospitalization Past 5 Years	NA	0.744	0.229	0.523	0.323	NA	0.534	0.85	0.722	NA	0.523	0.011
Life Expectancy	NA	0.717	0.991	0.945	0.883	NA	0.952	0.664	0.155	NA	0.007	0.189
Now Feeling Physically Miserable	NA	0.03	0.549	0.051	0.496	NA	0.786	0.834	0.211	NA	0.22	0.431
Usually Feeling Physically Miserable	NA	0.414	0.512	0.821	0.693	NA	0.17	0.208	0.439	NA	0.637	0.14
Now Feeling Mentally Miserable	NA	0.379	0.289	0.113	0.611	NA	0.348	0.796	0.415	NA	0.655	0.939
Usually Feeling Mentally Miserable	NA	0.402	0.903	0.808	0.783	NA	0.108	0.049	0.383	NA	0.75	0.824
Year	NA	NA	NA	NA	NA	NA	NA	NA	NA	NA	NA	NA
Course of COVID-19	NA	NA	0.716	0.373	0.786	NA	0.171	0.577	0.196	NA	0.252	0.709
Months since COVID Infection	NA	0.716	NA	0.848	0.29	NA	0.001	0	0.045	NA	0.688	0.666

Significant correlations are bolded. The p-values that remained or turned significant after the application of the Benjamini-Hochberg correction for multiple testing (with FDR set at 0.1) are underlined.

Table S16- Correlations between health and performance-related variables and COVID-related variables controlled for age, sex, and survey year- Women with at least 36 months elapsed since infection

	Infected	Course	Months since Infection	Vaccination	Months since Vaccination	Covid after Vaccination	Ancestral SARS-CoV-2 variant	Alpha	Delta	Omicron	Age	Sex
Physical Health Issues	NA	**0.373**	0.077	-0.1	0.072	NA	0.197	-0.135	-0.064	NA	-0.094	NA
Mental Health Issues	NA	0.124	0.213	-0.087	0.213	NA	-0.047	-0.02	-0.087	NA	-0.073	NA
Fatigue	NA	0.143	0.083	**0.228**	-0.107	NA	0.204	**-0.23**	0.01	NA	0.062	NA
Intelligence	NA	-0.103	-0.079	-0.205	-0.058	NA	0.15	-0.036	-0.013	NA	-0.158	NA
Memory	NA	-0.149	0.038	**-0.244**	-0.084	NA	-0.129	-0.108	0.202	NA	-0.117	NA
Reactions	NA	0.125	0.054	**-0.264**	0.013	NA	0.027	-0.104	0.044	NA	-0.085	NA
Accuracy	NA	0.032	0.144	-0.221	-0.001	NA	0.167	-0.118	-0.153	NA	-0.186	NA
Evolutionary Biology Score	NA	0.137	0.039	-0.028	0.09	NA	0.001	0.073	-0.15	NA	-0.24	NA
Free-Recall Memory	NA	-0.115	-0.009	-0.191	-0.04	NA	-0.166	-0.123	0.253	NA	-0.112	NA
Recognition Memory	NA	-0.113	0.025	-0.2	-0.076	NA	-0.092	-0.19	**0.245**	NA	-0.111	NA
Simple RT Duration	NA	0.161	**0.271**	**-0.261**	0.226	NA	0.102	-0.134	**-0.203**	NA	-0.012	NA
Simple RT Precision	NA	-0.066	0.098	-0.067	NA	NA	-0.146	0.109	0.038	NA	0.005	NA
Stroop Duration	NA	0.043	-0.077	0.001	0.054	NA	-0.055	-0.012	0.152	NA	-0.112	NA
Stroop Precision	NA	0.036	**0.386**	-0.111	0.074	NA	**0.427**	**-0.418**	**-0.379**	NA	-0.023	NA
Reading Time	NA	0.012	0.068	-0.033	0.103	NA	-0.04	-0.049	0.02	NA	0.055	NA
Allergy	NA	0.204	-0.114	0.028	0.005	NA	0.038	0.101	0.07	NA	-0.105	NA
Skin Problem	NA	**0.242**	0.054	-0.13	0.129	NA	0.025	-0.058	0.124	NA	-0.05	NA
Digestive Problem	NA	0.051	-0.1	-0.018	0.266	NA	0.121	-0.042	-0.012	NA	**0.227**	NA
Infection	NA	**0.228**	0.156	-0.206	-0.173	NA	**0.264**	**-0.223**	0.115	NA	-0.002	NA
Cardiovascular Problem	NA	-0.051	0.167	0.072	-0.332	NA	0.154	-0.113	-0.04	NA	-0.062	NA
Low Blood Pressure	NA	-0.184	0.071	-0.24	0.223	NA	-0.047	0.169	**-0.249**	NA	**-0.367**	NA
High Blood Pressure	NA	-0.179	-0.021	NA	-0.4	NA	-0.204	**0.423**	-0.117	NA	-0.042	NA
Orthopedic Problem	NA	-0.015	0.01	0.003	0.069	NA	0.073	0.052	**-0.222**	NA	0.013	NA
Metabolic Problem	NA	**0.244**	0.028	-0.073	**0.364**	NA	0.021	-0.08	-0.076	NA	0.096	NA
Neurologic Problem	NA	**0.248**	0.121	-0.1	-0.038	NA	0.197	-0.092	-0.083	NA	-0.088	NA
Headache	NA	0.171	0.189	0.098	-0.011	NA	0.079	-0.197	0.063	NA	**-0.285**	NA
Other Pain	NA	0.111	0.093	**-0.256**	0.141	NA	**0.291**	-0.212	-0.074	NA	**-0.255**	NA
Recurrent Problem	NA	0.062	-0.069	0.054	0.067	NA	0.211	-0.043	0	NA	-0.012	NA
Depression	NA	0.171	**0.24**	0.027	0.12	NA	0.058	-0.134	0.017	NA	0.078	NA
Anxiety	NA	0.152	0.163	-0.08	0.087	NA	0.164	-0.05	-0.05	NA	-0.028	NA
Other Psychological Problems	NA	**0.265**	0.035	-0.137	0.338	NA	0.1	-0.073	0.051	NA	-0.192	NA

Medication for Mental Purposes	NA	NA	NA	NA	NA	NA	NA	NA	NA	NA	NA	NA
Doctor Visits	NA	0.117	-0.147	-0.148	**-0.372**	NA	0.165	-0.097	**0.245**	NA	0.051	NA
Other Medication	NA	0.143	0.092	0.155	0.128	NA	-0.029	-0.023	-0.086	NA	-0.007	NA
Antibiotic Taken Past 3 Years	NA	0.122	-0.092	0.213	-0.271	NA	0.069	-0.032	**0.24**	NA	-0.066	NA
Hospitalization Past 5 Years	NA	-0.003	-0.01	0.089	-0.079	NA	0.191	-0.14	-0.05	NA	-0.071	NA
Life Expectancy	NA	0.085	-0.03	0.001	0.064	NA	-0.013	-0.054	0.169	NA	-0.192	NA
Now Feeling Physically Miserable	NA	**0.224**	0.182	-0.218	0.122	NA	0.105	-0.102	-0.146	NA	**-0.231**	NA
Usually Feeling Physically Miserable	NA	0.093	-0.065	0.026	0.016	NA	-0.128	0.115	-0.085	NA	-0.014	NA
Now Feeling Mentally Miserable	NA	0.026	**0.302**	-0.175	0.243	NA	0.003	-0.168	-0.1	NA	-0.05	NA
Usually Feeling Mentally Miserable	NA	0.175	0.118	-0.028	0.111	NA	-0.069	0.102	-0.105	NA	-0.018	NA
Year	NA	NA	NA	NA	NA	NA	NA	NA	NA	NA	NA	NA
Course of COVID-19	NA	NA	0.079	-0.095	-0.017	NA	0.203	-0.113	-0.14	NA	0.081	NA
Months since COVID Infection	NA	0.079	NA	-0.03	0.088	NA	**0.289**	**-0.514**	**-0.225**	NA	0.062	NA

Significant correlations are bolded. The p-values that remained or turned significant after the application of the Benjamini-Hochberg correction for multiple testing (with FDR set at 0.1) are underlined.

Table S17- P-values of the correlations between health and performance-related variables and COVID-related variables controlled for age, sex, and survey year- women with at least 36 months elapsed since infection

	Infected	Course	Months since Infection	Vaccination	Months since Vaccination	Covid after Vaccination	Ancestral SARS-CoV-2 variant	Alpha	Delta	Omicron	Age	Sex
Physical Health Issues	NA	0.001	0.499	0.378	0.678	NA	0.082	0.234	0.574	NA	0.401	NA
Mental Health Issues	NA	0.272	0.059	0.442	0.218	NA	0.68	0.862	0.444	NA	0.515	NA
Fatigue	NA	0.207	0.463	0.044	0.536	NA	0.072	0.042	0.931	NA	0.581	NA
Intelligence	NA	0.364	0.484	0.07	0.738	NA	0.186	0.752	0.907	NA	0.157	NA
Memory	NA	0.188	0.739	0.031	0.627	NA	0.256	0.339	0.074	NA	0.296	NA
Reactions	NA	0.267	0.632	0.02	0.938	NA	0.815	0.356	0.694	NA	0.448	NA
Accuracy	NA	0.778	0.203	0.051	0.995	NA	0.139	0.298	0.175	NA	0.095	NA
Evolutionary Biology Score	NA	0.231	0.734	0.806	0.601	NA	0.994	0.523	0.19	NA	0.034	NA
Free-Recall Memory	NA	0.346	0.94	0.119	0.822	NA	0.175	0.314	0.039	NA	0.353	NA
Recognition Memory	NA	0.338	0.835	0.09	0.661	NA	0.436	0.109	0.039	NA	0.339	NA
Simple RT Duration	NA	0.154	0.016	0.021	0.191	NA	0.365	0.238	0.072	NA	0.914	NA
Simple RT Precision	NA	0.557	0.389	0.552	19	NA	0.196	0.337	0.739	NA	0.966	NA
Stroop Duration	NA	0.701	0.494	0.99	0.755	NA	0.626	0.913	0.18	NA	0.316	NA
Stroop Precision	NA	0.752	0.001	0.336	0.667	NA	0	0	0.001	NA	0.842	NA
Reading Time	NA	0.916	0.549	0.771	0.549	NA	0.721	0.667	0.857	NA	0.622	NA
Allergy	NA	0.072	0.314	0.805	0.978	NA	0.736	0.371	0.538	NA	0.346	NA
Skin Problem	NA	0.033	0.633	0.252	0.456	NA	0.822	0.607	0.272	NA	0.656	NA
Digestive Problem	NA	0.652	0.377	0.876	0.123	NA	0.283	0.71	0.919	NA	0.041	NA
Infection	NA	0.044	0.169	0.069	0.316	NA	0.02	0.049	0.307	NA	0.986	NA
Cardiovascular Problem	NA	0.65	0.14	0.524	0.055	NA	0.173	0.317	0.722	NA	0.577	NA
Low Blood Pressure	NA	0.14	0.568	0.054	0.229	NA	0.708	0.174	0.045	NA	0.003	NA
High Blood Pressure	NA	0.371	0.918	2	0.259	NA	0.308	0.035	0.559	NA	0.829	NA
Orthopedic Problem	NA	0.892	0.933	0.98	0.688	NA	0.521	0.644	0.049	NA	0.905	NA
Metabolic Problem	NA	0.031	0.805	0.522	0.035	NA	0.854	0.479	0.502	NA	0.391	NA
Neurologic Problem	NA	0.028	0.285	0.377	0.824	NA	0.082	0.415	0.461	NA	0.429	NA
Headache	NA	0.13	0.095	0.384	0.95	NA	0.486	0.082	0.576	NA	0.011	NA
Other Pain	NA	0.325	0.411	0.024	0.414	NA	0.01	0.061	0.513	NA	0.022	NA
Recurrent Problem	NA	0.584	0.544	0.636	0.7	NA	0.063	0.705	0.997	NA	0.914	NA
Depression	NA	0.132	0.034	0.808	0.485	NA	0.608	0.237	0.879	NA	0.484	NA
Anxiety	NA	0.178	0.149	0.479	0.615	NA	0.146	0.656	0.661	NA	0.801	NA
Other Psychological Problems	NA	0.019	0.755	0.226	0.05	NA	0.379	0.518	0.655	NA	0.084	NA

Medication for Mental Purposes	NA	NA	NA	NA	NA	NA	NA	NA	NA	NA	NA	NA
Doctor Visits	NA	0.302	0.194	0.191	0.031	NA	0.144	0.393	0.031	NA	0.644	NA
Other Medication	NA	0.243	0.451	0.205	0.474	NA	0.811	0.85	0.48	NA	0.956	NA
Antibiotic Taken Past 3 Years	NA	0.28	0.416	0.06	0.116	NA	0.54	0.776	0.034	NA	0.552	NA
Hospitalization Past 5 Years	NA	0.981	0.931	0.431	0.645	NA	0.092	0.215	0.659	NA	0.523	NA
Life Expectancy	NA	0.454	0.792	0.993	0.709	NA	0.907	0.632	0.135	NA	0.086	NA
Now Feeling Physically Miserable	NA	0.047	0.108	0.054	0.478	NA	0.352	0.369	0.196	NA	0.038	NA
Usually Feeling Physically Miserable	NA	0.412	0.568	0.817	0.928	NA	0.259	0.309	0.452	NA	0.898	NA
Now Feeling Mentally Miserable	NA	0.82	0.008	0.121	0.159	NA	0.975	0.137	0.375	NA	0.655	NA
Usually Feeling Mentally Miserable	NA	0.122	0.298	0.807	0.522	NA	0.541	0.367	0.354	NA	0.875	NA
Year	NA	NA	NA	NA	NA	NA	NA	NA	NA	NA	NA	NA
Course of COVID-19	NA	NA	0.484	0.399	0.922	NA	0.073	0.32	0.217	NA	0.466	NA
Months since COVID Infection	NA	0.484	NA	0.794	0.611	NA	0.011	0	0.047	NA	0.578	NA

Significant correlations are bolded. The p-values that remained or turned significant after the application of the Benjamini-Hochberg correction for multiple testing (with FDR set at 0.1) are underlined.

Table S18- Correlations between health and performance-related variables and COVID-related variables controlled for age, sex, and survey year- men with at least 36 months elapsed since infection

	Infected	Course	Months since Infection	Vaccination	Months since Vaccination	Covid after Vaccination	Ancestral SARS-CoV-2 variant	Alpha	Delta	Omicron	Age	Sex
Physical Health Issues	NA	0.232	-0.328	NA	-0.055	NA	-0.426	0.426	NA	NA	-0.144	NA
Mental Health Issues	NA	0.004	-0.414	NA	-0.464	NA	-0.389	0.389	NA	NA	-0.016	NA
Fatigue	NA	-0.341	-0.159	NA	-0.706	NA	-0.017	0.017	NA	NA	0.094	NA
Intelligence	NA	0.165	0.129	NA	0.468	NA	-0.057	0.057	NA	NA	0.307	NA
Memory	NA	-0.241	0.314	NA	0.342	NA	0.36	-0.36	NA	NA	0.394	NA
Reactions	NA	-0.056	0.35	NA	0.196	NA	**0.534**	**-0.534**	NA	NA	0.208	NA
Accuracy	NA	0.065	0.06	NA	-0.342	NA	-0.359	0.359	NA	NA	-0.24	NA
Evolutionary Biology Score	NA	-0.004	-0.091	NA	-0.148	NA	-0.258	0.258	NA	NA	**-0.504**	NA
Free-Recall Memory	NA	-0.259	0.178	NA	0.274	NA	0.221	-0.221	NA	NA	0.289	NA
Recognition Memory	NA	-0.309	0.281	NA	0.274	NA	0.35	-0.35	NA	NA	0.379	NA
Simple RT Duration	NA	-0.107	0.159	NA	0.071	NA	0.205	-0.205	NA	NA	0.112	NA
Simple RT Precision	NA	NA	NA	NA	NA	NA	NA	NA	NA	NA	NA	NA
Stroop Duration	NA	-0.218	0.207	NA	-0.189	NA	0.288	-0.288	NA	NA	-0.048	NA
Stroop Precision	NA	NA	NA	NA	NA	NA	NA	NA	NA	NA	NA	NA
Reading Time	NA	-0.203	0.371	NA	0.189	NA	**0.479**	**-0.479**	NA	NA	0.176	NA
Allergy	NA	0.202	**-0.666**	NA	-0.508	NA	**-0.52**	**0.52**	NA	NA	0.124	NA
Skin Problem	NA	-0.027	-0.183	NA	-0.225	NA	-0.165	0.165	NA	NA	-0.401	NA
Digestive Problem	NA	-0.016	0.071	NA	-0.76	NA	-0.232	0.232	NA	NA	-0.407	NA
Infection	NA	0.094	-0.197	NA	-0.286	NA	-0.361	0.361	NA	NA	-0.154	NA
Cardiovascular Problem	NA	0.2	**-0.478**	NA	NA	NA	**-0.665**	**0.665**	NA	NA	-0.353	NA
Low Blood Pressure	NA	-0.03	-0.052	NA	0.582	NA	0.166	-0.166	NA	NA	0.34	NA
High Blood Pressure	NA	NA	NA	NA	NA	NA	NA	NA	NA	NA	NA	NA
Orthopedic Problem	NA	0.053	0.112	NA	-0.019	NA	-0.037	0.037	NA	NA	0.035	NA
Metabolic Problem	NA	0.414	-0.2	NA	NA	NA	0.092	-0.092	NA	NA	0.275	NA
Neurologic Problem	NA	NA	NA	NA	NA	NA	NA	NA	NA	NA	NA	NA
Headache	NA	0.054	-0.219	NA	-0.141	NA	-0.393	0.393	NA	NA	-0.201	NA
Other Pain	NA	0.096	-0.252	NA	0.183	NA	-0.363	0.363	NA	NA	-0.266	NA
Recurrent Problem	NA	-0.096	-0.084	NA	-0.292	NA	-0.407	0.407	NA	NA	**-0.506**	NA
Depression	NA	-0.247	-0.337	NA	-0.605	NA	-0.449	0.449	NA	NA	-0.125	NA
Anxiety	NA	-0.282	-0.39	NA	-0.499	NA	-0.331	0.331	NA	NA	-0.072	NA
Other Psychological Problems	NA	-0.13	-0.401	NA	-0.299	NA	-0.212	0.212	NA	NA	0.276	NA

Medication for Mental Purposes	NA	NA	NA	NA	NA	NA	NA	NA	NA	NA	NA	NA
Doctor Visits	NA	0.351	-0.095	NA	0.183	NA	-0.407	0.407	NA	NA	-0.122	NA
Other Medication	NA	0.44	-0.474	NA	NA	NA	-0.384	0.384	NA	NA	-0.067	NA
Antibiotic Taken Past 3 Years	NA	0.36	0.054	NA	0.299	NA	-0.002	0.002	NA	NA	**-0.519**	NA
Hospitalization Past 5 Years	NA	0.104	-0.301	NA	-0.109	NA	-0.154	0.154	NA	NA	-0.071	NA
Life Expectancy	NA	-0.079	0.026	NA	-0.327	NA	0.065	-0.065	NA	NA	**-0.502**	NA
Now Feeling Physically Miserable	NA	0.22	-0.253	NA	0.41	NA	-0.238	0.238	NA	NA	0.168	NA
Usually Feeling Physically Miserable	NA	0.055	-0.189	NA	0.2	NA	-0.186	0.186	NA	NA	-0.118	NA
Now Feeling Mentally Miserable	NA	0.273	**-0.493**	NA	-0.334	NA	**-0.473**	**0.473**	NA	NA	0.281	NA
Usually Feeling Mentally Miserable	NA	-0.151	-0.404	NA	-0.612	NA	-0.452	0.452	NA	NA	-0.071	NA
Year	NA	NA	NA	NA	NA	NA	NA	NA	NA	NA	NA	NA
Course of COVID-19	NA	NA	-0.045	NA	0.422	NA	-0.188	0.188	NA	NA	0.255	NA
Months since COVID Infection	NA	-0.045	NA	NA	0.378	NA	**0.631**	**-0.631**	NA	NA	-0.272	NA

Significant correlations are bolded. The p-values that remained or turned significant after the application of the Benjamini-Hochberg correction for multiple testing (with FDR set at 0.1) are underlined.

Table S19- P-values of the correlations between health and performance-related variables and COVID-related variables controlled for age, sex, and survey year- men with at least 36 months elapsed since infection

	Infected	Course	Months since Infection	Vaccination	Months since Vaccination	Covid after Vaccination	Ancestral SARS-CoV-2 variant	Alpha	Delta	Omicron	Age	Sex
Physical Health Issues	NA	0.32	0.161	NA	0.894	NA	0.068	0.068	NA	NA	0.514	NA
Mental Health Issues	NA	0.986	0.076	NA	0.255	NA	0.096	0.096	NA	NA	0.942	NA
Fatigue	NA	0.144	0.497	NA	0.084	NA	0.943	0.943	NA	NA	0.671	NA
Intelligence	NA	0.48	0.582	NA	0.252	NA	0.807	0.807	NA	NA	0.165	NA
Memory	NA	0.302	0.179	NA	0.402	NA	0.123	0.123	NA	NA	0.075	NA
Reactions	NA	0.81	0.134	NA	0.63	NA	0.022	0.022	NA	NA	0.346	NA
Accuracy	NA	0.78	0.798	NA	0.402	NA	0.124	0.124	NA	NA	0.277	NA
Evolutionary Biology Score	NA	0.987	0.696	NA	0.717	NA	0.269	0.269	NA	NA	0.022	NA
Free-Recall Memory	NA	0.297	0.473	NA	0.503	NA	0.375	0.375	NA	NA	0.216	NA
Recognition Memory	NA	0.186	0.229	NA	0.503	NA	0.134	0.134	NA	NA	0.086	NA
Simple RT Duration	NA	0.647	0.497	NA	0.861	NA	0.38	0.38	NA	NA	0.612	NA
Simple RT Precision	NA	NA	NA	NA	NA	NA	NA	NA	NA	NA	NA	NA
Stroop Duration	NA	0.351	0.375	NA	0.643	NA	0.217	0.217	NA	NA	0.828	NA
Stroop Precision	NA	NA	NA	NA	NA	NA	NA	NA	NA	NA	NA	NA
Reading Time	NA	0.384	0.112	NA	0.643	NA	0.04	0.04	NA	NA	0.425	NA
Allergy	NA	0.387	0.004	NA	0.213	NA	0.026	0.026	NA	NA	0.575	NA
Skin Problem	NA	0.906	0.434	NA	0.582	NA	0.479	0.479	NA	NA	0.07	NA
Digestive Problem	NA	0.946	0.762	NA	0.063	NA	0.321	0.321	NA	NA	0.066	NA
Infection	NA	0.686	0.398	NA	0.484	NA	0.122	0.122	NA	NA	0.486	NA
Cardiovascular Problem	NA	0.391	0.041	NA	6	NA	0.004	0.004	NA	NA	0.11	NA
Low Blood Pressure	NA	0.903	0.834	NA	0.236	NA	0.504	0.504	NA	NA	0.145	NA
High Blood Pressure	NA	NA	NA	NA	NA	NA	NA	NA	NA	NA	NA	NA
Orthopedic Problem	NA	0.819	0.63	NA	0.963	NA	0.876	0.876	NA	NA	0.875	NA
Metabolic Problem	NA	0.077	0.392	NA	6	NA	0.694	0.694	NA	NA	0.214	NA
Neurologic Problem	NA	NA	NA	NA	NA	NA	NA	NA	NA	NA	NA	NA
Headache	NA	0.817	0.348	NA	0.73	NA	0.093	0.093	NA	NA	0.363	NA
Other Pain	NA	0.68	0.28	NA	0.654	NA	0.12	0.12	NA	NA	0.229	NA
Recurrent Problem	NA	0.681	0.72	NA	0.474	NA	0.081	0.081	NA	NA	0.022	NA
Depression	NA	0.29	0.149	NA	0.138	NA	0.054	0.054	NA	NA	0.571	NA
Anxiety	NA	0.228	0.095	NA	0.222	NA	0.156	0.156	NA	NA	0.746	NA
Other Psychological Problems	NA	0.601	0.107	NA	0.464	NA	0.394	0.394	NA	NA	0.237	NA

Medication for Mental Purposes	NA	NA	NA	NA	NA	NA	NA	NA	NA	NA	NA	NA
Doctor Visits	NA	0.132	0.684	NA	0.654	NA	0.081	0.081	NA	NA	0.581	NA
Other Medication	NA	0.076	0.057	NA	6	NA	0.122	0.122	NA	NA	0.775	NA
Antibiotic Taken Past 3 Years	NA	0.123	0.816	NA	0.464	NA	0.994	0.994	NA	NA	0.019	NA
Hospitalization Past 5 Years	NA	0.655	0.197	NA	0.79	NA	0.511	0.511	NA	NA	0.747	NA
Life Expectancy	NA	0.737	0.912	NA	0.423	NA	0.779	0.779	NA	NA	0.023	NA
Now Feeling Physically Miserable	NA	0.345	0.278	NA	0.316	NA	0.307	0.307	NA	NA	0.447	NA
Usually Feeling Physically Miserable	NA	0.814	0.419	NA	0.623	NA	0.426	0.426	NA	NA	0.594	NA
Now Feeling Mentally Miserable	NA	0.242	0.035	NA	0.413	NA	0.043	0.043	NA	NA	0.203	NA
Usually Feeling Mentally Miserable	NA	0.517	0.084	NA	0.134	NA	0.053	0.053	NA	NA	0.748	NA
Year	NA	NA	NA	NA	NA	NA	NA	NA	NA	NA	NA	NA
Course of COVID-19	NA	NA	0.847	NA	0.301	NA	0.42	0.42	NA	NA	0.248	NA
Months since COVID Infection	NA	0.847	NA	NA	0.355	NA	0.007	0.007	NA	NA	0.218	NA

Significant correlations are bolded. The p-values that remained or turned significant after the application of the Benjamini-Hochberg correction for multiple testing (with FDR set at 0.1) are underlined.

Table S20- Correlations between health and performance-related variables and COVID-related variables controlled for age, sex, and survey year- All subjects

	Infected	Course	Months since Infection	Vaccination	Months since Vaccination	Covid after Vaccination	Ancestral SARS-CoV-2 variant	Alpha	Delta	Omicron	Age	Sex
Physical Health Issues	0.012	**0.211**	0.013	**0.062**	0.013	-0.012	0.05	-0.024	0.001	-0.029	0.012	**0.163**
Mental Health Issues	-0.049	0.066	-0.045	0.048	-0.015	-0.025	-0.051	0.018	-0.023	0.052	0.036	**0.162**
Fatigue	-0.042	**0.093**	0.044	**0.089**	0.036	-0.005	**0.126**	**-0.103**	-0.062	-0.007	-0.005	**0.097**
Intelligence	0.021	-0.057	-0.071	-0.044	0.005	**0.171**	**-0.099**	-0.076	0.018	**0.172**	-0.021	**-0.099**
Memory	0.045	-0.01	-0.053	-0.039	-0.011	0.029	**-0.119**	-0.035	0.065	0.068	**-0.055**	**0.111**
Reactions	0.029	0.029	0.036	**-0.075**	0.027	-0.067	**0.097**	**-0.095**	-0.025	-0.024	-0.009	0.02
Accuracy	0.001	**-0.082**	-0.025	-0.015	0.051	**0.194**	-0.023	-0.073	0.01	0.075	-0.053	**-0.096**
Evolutionary Biology Score	-0.014	0.009	0.028	0.025	0.065	**0.117**	0.001	0.052	0.011	-0.047	**-0.099**	**-0.073**
Free-Recall Memory	0.053	-0.017	-0.063	-0.004	-0.014	0.063	**-0.095**	-0.043	0.052	0.057	-0.052	**0.108**
Recognition Memory	**0.057**	-0.018	-0.063	-0.012	-0.011	0.065	**-0.094**	-0.075	0.081	0.055	-0.043	**0.117**
Simple RT Duration	0.005	-0.068	0.041	**-0.058**	0.043	0.009	0.066	-0.048	-0.037	-0.018	0.035	0.046
Simple RT Precision	0.007	-0.009	-0.018	-0.05	0.045	0.014	0.019	**-0.194**	0.067	0.054	-0.008	**-0.074**
Stroop Duration	0.015	0.04	0.036	-0.051	0.013	-0.043	0.083	-0.069	0.016	-0.062	0.001	-0.002
Stroop Precision	-0.006	**-0.095**	-0.019	-0.011	0.044	**0.12**	0.041	-0.042	-0.07	0.043	-0.016	-0.008
Reading Time	0.041	-0.011	0.044	-0.049	-0.01	-0.073	0.055	-0.053	-0.017	-0.027	-0.002	0.053
Allergy	0.017	**0.128**	0.009	**0.067**	0.019	0.022	0.041	0.001	0.022	-0.045	-0.006	**0.07**
Skin Problem	0.016	**0.164**	0.002	0.016	0.026	**0.128**	0.013	-0.055	0.032	0.001	-0.03	-0.018
Digestive Problem	-0.003	**0.125**	-0.02	**0.064**	0.04	-0.052	0.001	0.04	-0.04	0.024	0.026	0.004
Infection	**0.101**	**0.189**	-0.001	0.03	-0.016	0.065	0.06	**-0.127**	0.017	0.037	**-0.055**	**0.055**
Cardiovascular Problem	0.017	0.004	0.03	**0.075**	-0.033	-0.014	0.051	-0.01	-0.063	0.019	-0.029	0.026
Low Blood Pressure	-0.046	0.02	0.064	0.043	0.048	-0.103	0.067	0.012	-0.015	-0.078	-0.013	**0.254**
High Blood Pressure	-0.06	0	0.022	0.034	0.089	**0.16**	-0.053	-0.006	0.084	-0.01	**0.086**	-0.034
Orthopedic Problem	0.026	0.046	0.037	0.048	0.024	-0.01	0.02	**0.108**	-0.031	-0.08	0.046	**0.159**
Metabolic Problem	0.04	0.043	0.027	-0.017	-0.018	-0.002	0.003	-0.01	0.064	-0.065	0.021	**0.096**
Neurologic Problem	**0.061**	**0.125**	-0.033	-0.043	0.026	0.074	0	-0.058	0.015	0.044	0.012	**0.079**
Headache	-0.005	**0.13**	0.015	**0.109**	-0.025	0	0.012	0.047	-0.021	-0.041	-0.043	**0.161**
Other Pain	-0.02	**0.141**	0.022	0.005	0.004	0.01	0.072	-0.015	-0.004	-0.056	-0.028	**0.089**
Recurrent Problem	0.027	**0.08**	0.019	**0.056**	-0.012	0.053	**0.125**	-0.017	-0.085	-0.017	0.031	**0.07**
Depression	0.008	**0.088**	-0.084	0.014	-0.014	-0.054	-0.074	-0.029	-0.029	**0.128**	**0.069**	**0.081**
Anxiety	-0.008	0.07	-0.053	0.046	0.002	0.071	-0.008	-0.06	-0.054	**0.12**	0.035	**0.156**
Other Psychological Problems	-0.033	**0.115**	0.027	0.026	-0.001	-0.033	0.035	0.004	-0.05	0.013	0.008	**0.072**

Medication for Mental Purposes	-0.022	**0.155**	-0.074	0.045	0.003	-0.04	-0.078	-0.026	0.036	0.077	0.035	**0.118**
Doctor Visits	0.046	**0.144**	-0.085	0.024	0.016	-0.014	-0.043	0.049	-0.038	0.08	-0.05	-0.009
Other Medication	-0.052	**0.186**	-0.024	**0.118**	0	0.084	0.017	-0.069	-0.044	0.059	0.004	**0.137**
Antibiotic Taken Past 3 Years	**0.063**	**0.099**	-0.01	0.002	0.034	0.013	-0.014	-0.014	0.022	0.03	-0.016	**0.117**
Hospitalization Past 5 Years	0.019	**0.125**	0.017	-0.005	0.001	-0.082	0.05	0.002	0.022	-0.071	-0.028	**-0.059**
Life Expectancy	-0.016	**0.096**	0.058	0.037	-0.024	**-0.128**	0.011	-0.013	0.06	-0.063	-0.021	-0.038
Now Feeling Physically Miserable	-0.007	0.074	-0.002	0.016	-0.007	0.02	-0.008	-0.01	-0.016	0.027	**0.074**	**0.103**
Usually Feeling Physically Miserable	-0.039	0.05	-0.061	0.029	-0.047	-0.022	-0.07	-0.009	0.036	0.038	0.029	**0.17**
Now Feeling Mentally Miserable	-0.022	0.053	0.001	-0.006	-0.028	-0.011	-0.017	0.01	0.024	-0.043	0.043	**0.129**
Usually Feeling Mentally Miserable	**-0.088**	0.034	-0.068	**0.071**	-0.015	-0.097	-0.072	**0.116**	-0.037	0.027	0.021	**0.149**
Year	**0.37**	**0.106**	**0.618**	**-0.096**	**0.71**	0.09	**-0.148**	**-0.132**	-0.018	**0.248**	0.018	0.05
Course of COVID-19	NA	NA	-0.01	-0.037	-0.007	0.032	0.033	-0.034	-0.001	0.001	**0.101**	0.05
Months since COVID Infection	-0.036	-0.01	NA	0.032	**0.144**	**-0.405**	**0.47**	0.032	**-0.103**	**-0.479**	0.007	-0.034

Sex (last column) was coded as 1 – men and 2 – women. Significant correlations are bolded. The p-values that remained or turned significant after the application of the Benjamini-Hochberg correction for multiple testing (with FDR set at 0.1) are underlined.

Table S21- P-values of the correlations between health and performance-related variables and COVID-related variables controlled for age, sex, and survey year- All subjects

	Infected	Course	Months since Infection	Vaccination	Months since Vaccination	Covid after Vaccination	Ancestral SARS-CoV-2 variant	Alpha	Delta	Omicron	Age	Sex
Physical Health Issues	0.655	0	0.769	0.026	0.707	0.828	0.251	0.585	0.988	0.51	0.672	0
Mental Health Issues	0.079	0.101	0.298	0.084	0.67	0.653	0.245	0.675	0.601	0.237	0.191	0
Fatigue	0.128	0.021	0.313	0.002	0.314	0.931	0.004	0.018	0.159	0.881	0.846	0.001
Intelligence	0.451	0.161	0.106	0.122	0.887	0.002	0.024	0.083	0.676	0	0.451	0
Memory	0.108	0.809	0.227	0.167	0.757	0.612	0.007	0.428	0.141	0.119	0.048	0
Reactions	0.302	0.473	0.411	0.007	0.444	0.23	0.027	0.03	0.565	0.584	0.744	0.467
Accuracy	0.973	0.041	0.566	0.587	0.145	0.001	0.599	0.093	0.812	0.088	0.057	0.001
Evolutionary Biology Score	0.615	0.828	0.521	0.365	0.064	0.037	0.989	0.237	0.799	0.283	0	0.009
Free-Recall Memory	0.071	0.68	0.166	0.892	0.693	0.279	0.038	0.348	0.252	0.213	0.076	0
Recognition Memory	0.048	0.661	0.172	0.672	0.762	0.257	0.04	0.1	0.077	0.233	0.139	0
Simple RT Duration	0.858	0.089	0.346	0.039	0.223	0.874	0.133	0.268	0.393	0.673	0.213	0.1
Simple RT Precision	0.794	0.821	0.686	0.072	0.206	0.809	0.67	0	0.125	0.217	0.782	0.007
Stroop Duration	0.587	0.318	0.409	0.067	0.718	0.442	0.057	0.115	0.718	0.159	0.972	0.933
Stroop Precision	0.817	0.019	0.657	0.686	0.217	0.032	0.348	0.341	0.111	0.323	0.567	0.775
Reading Time	0.144	0.778	0.313	0.082	0.771	0.19	0.212	0.226	0.693	0.535	0.941	0.056
Allergy	0.545	0.001	0.83	0.016	0.582	0.687	0.344	0.991	0.622	0.299	0.821	0.012
Skin Problem	0.573	0	0.957	0.573	0.458	0.022	0.769	0.207	0.471	0.989	0.28	0.522
Digestive Problem	0.903	0.002	0.645	0.023	0.254	0.354	0.98	0.358	0.36	0.588	0.35	0.875
Infection	0	0	0.982	0.284	0.65	0.243	0.174	0.004	0.696	0.397	0.047	0.047
Cardiovascular Problem	0.548	0.915	0.489	0.007	0.343	0.798	0.239	0.82	0.151	0.664	0.294	0.351
Low Blood Pressure	0.138	0.653	0.183	0.167	0.216	0.096	0.165	0.804	0.751	0.106	0.685	0
High Blood Pressure	0.134	0.998	0.712	0.413	0.078	0.041	0.381	0.924	0.166	0.871	0.032	0.402
Orthopedic Problem	0.354	0.248	0.395	0.085	0.497	0.852	0.653	0.013	0.476	0.069	0.094	0
Metabolic Problem	0.157	0.287	0.54	0.553	0.614	0.969	0.951	0.824	0.145	0.138	0.442	0.001
Neurologic Problem	0.029	0.002	0.455	0.129	0.459	0.187	0.991	0.183	0.737	0.319	0.665	0.005
Headache	0.853	0.001	0.73	0	0.479	0.993	0.79	0.286	0.625	0.347	0.122	0
Other Pain	0.484	0	0.622	0.859	0.901	0.863	0.099	0.738	0.923	0.2	0.311	0.001
Recurrent Problem	0.33	0.048	0.67	0.045	0.741	0.341	0.004	0.692	0.053	0.698	0.273	0.012
Depression	0.775	0.029	0.054	0.623	0.691	0.329	0.091	0.508	0.501	0.003	0.013	0.003
Anxiety	0.773	0.084	0.221	0.104	0.95	0.202	0.85	0.17	0.215	0.006	0.214	0
Other Psychological Problems	0.233	0.005	0.543	0.355	0.976	0.561	0.42	0.929	0.252	0.761	0.762	0.01

Medication for Mental Purposes	0.449	0	0.104	0.126	0.939	0.491	0.086	0.576	0.426	0.09	0.224	0
Doctor Visits	0.098	0	0.052	0.397	0.643	0.8	0.33	0.265	0.39	0.068	0.074	0.741
Other Medication	0.073	0	0.592	0	0.991	0.143	0.704	0.131	0.335	0.191	0.885	0
Antibiotic Taken Past 3 Years	0.024	0.014	0.825	0.95	0.338	0.812	0.753	0.747	0.61	0.486	0.572	0
Hospitalization Past 5 Years	0.49	0.002	0.692	0.846	0.978	0.145	0.25	0.966	0.614	0.107	0.315	0.034
Life Expectancy	0.579	0.018	0.186	0.195	0.507	0.022	0.803	0.764	0.171	0.153	0.465	0.177
Now Feeling Physically Miserable	0.8	0.066	0.967	0.578	0.843	0.725	0.848	0.817	0.71	0.538	0.008	0
Usually Feeling Physically Miserable	0.158	0.213	0.165	0.299	0.183	0.696	0.111	0.829	0.415	0.381	0.301	0
Now Feeling Mentally Miserable	0.433	0.185	0.988	0.821	0.419	0.847	0.704	0.818	0.585	0.323	0.119	0
Usually Feeling Mentally Miserable	0.002	0.393	0.119	0.011	0.671	0.081	0.101	0.008	0.4	0.544	0.439	0
Year	0	0.008	0	0.001	0	0.107	0.001	0.003	0.688	0	0.51	0.073
Course of COVID-19	NA	NA	0.822	0.364	0.895	0.562	0.45	0.444	0.983	0.974	0.012	0.211
Months since COVID Infection	0.411	0.822	NA	0.476	0.01	0	0	0.465	0.018	0	0.877	0.431

Table S22- Correlations between health and performance-related variables and COVID-related variables controlled for age, sex, survey year, and time elapsed since infection- All subjects

	Infected	Course	Months since Infection	Vaccination	Months since Vaccination	Covid after Vaccination	Ancestral SARS-CoV-2 variant	Alpha	Delta	Omicron	Age	Sex
Physical Health Issues	0.015	**0.210**	0.017	**0.061**	0.013	-0.022	0.049	-0.024	0.001	-0.025	0.010	**0.161**
Mental Health Issues	-0.053	0.066	-0.022	0.048	-0.015	-0.054	-0.033	0.019	-0.027	0.034	0.037	**0.163**
Fatigue	-0.035	**0.092**	0.044	**0.088**	0.033	0.009	**0.119**	**-0.104**	-0.057	0.016	-0.008	**0.093**
Intelligence	0.014	-0.056	-0.036	-0.043	0.006	**0.162**	-0.075	-0.073	0.011	**0.157**	-0.019	**-0.096**
Memory	0.038	-0.009	-0.040	-0.038	-0.008	-0.003	**-0.106**	-0.032	0.059	0.048	-0.052	**0.113**
Reactions	0.029	0.028	0.001	**-0.075**	0.026	-0.042	**0.090**	**-0.096**	-0.021	-0.007	-0.009	0.020
Accuracy	0.008	**-0.082**	0.040	-0.015	0.050	**0.195**	-0.012	-0.072	0.007	0.071	**-0.055**	**-0.097**
Output Variables												
Evolutionary Biology Score	-0.000	0.008	**0.075**	0.024	0.062	**0.126**	-0.014	0.051	0.014	-0.038	**-0.103**	**-0.077**
Free-Recall Memory	0.044	-0.017	-0.049	-0.003	-0.011	0.026	-0.073	-0.040	0.046	0.030	-0.048	**0.110**
Recognition Memory	0.049	-0.018	-0.051	-0.011	-0.007	0.029	-0.073	-0.073	0.075	0.027	-0.038	**0.119**
Simple RT Duration	0.008	-0.068	0.019	**-0.058**	0.042	0.039	0.052	-0.049	-0.033	0.001	0.033	0.044
Simple RT Precision	0.008	-0.009	0.008	-0.050	0.043	0.027	0.030	**-0.193**	0.065	0.051	-0.008	**-0.074**
Stroop Duration	0.016	0.040	0.007	-0.051	0.012	-0.014	0.075	-0.070	0.019	-0.050	0.000	-0.002
Stroop Precision	-0.001	**-0.094**	0.026	-0.011	0.043	**0.110**	0.056	-0.041	-0.072	0.038	-0.017	-0.009
Reading Time	0.036	-0.011	-0.024	-0.048	-0.010	-0.062	0.038	-0.054	-0.012	-0.006	-0.000	**0.054**
Allergy	0.024	**0.127**	0.038	**0.066**	0.017	0.031	0.041	0.000	0.022	-0.046	-0.008	**0.067**
Skin Problem	0.015	**0.163**	-0.000	0.015	0.027	0.109	0.013	-0.055	0.031	0.001	-0.029	-0.017
Digestive Problem	-0.005	**0.125**	-0.012	**0.064**	0.040	-0.054	0.011	0.040	-0.042	0.015	0.026	0.005
Infection	**0.107**	**0.189**	0.028	0.029	-0.016	0.051	0.067	**-0.127**	0.017	0.041	**-0.056**	0.053
Cardiovascular Problem	0.023	0.004	0.038	**0.074**	-0.035	-0.004	0.042	-0.010	-0.059	0.038	-0.031	0.023
Low Blood Pressure	-0.036	0.021	0.055	0.0399	0.047	-0.112	0.041	0.011	-0.007	-0.054	-0.016	**0.250**
High Blood Pressure	-0.057	0.000	0.017	0.030	0.089	**0.180**	-0.073	-0.006	0.087	0.000	**0.085**	-0.035
Orthopedic Problem	0.026	0.046	0.004	0.048	0.023	-0.005	0.002	**0.107**	-0.027	-0.070	0.046	**0.158**
Metabolic Problem	0.041	0.042	0.007	-0.016	-0.016	-0.001	-0.011	-0.010	0.066	-0.059	0.020	**0.095**
Neurologic Problem	0.052	**0.125**	-0.050	-0.041	0.027	0.075	0.018	-0.057	0.011	0.031	0.015	**0.081**
Headache	0.000	**0.130**	0.028	**0.108**	-0.024	-0.018	0.005	0.046	-0.019	-0.038	-0.044	**0.158**
Other Pain	-0.016	**0.141**	0.020	0.004	0.004	0.013	0.070	-0.015	-0.002	-0.052	-0.029	**0.087**
Recurrent Problem	0.031	**0.079**	0.021	**0.055**	-0.011	0.052	**0.132**	-0.017	-0.083	-0.009	0.029	**0.068**
Depression	0.000	**0.088**	-0.044	0.014	-0.013	-0.091	-0.039	-0.026	-0.038	**0.100**	**0.071**	**0.083**
Anxiety	-0.011	0.069	-0.017	0.046	0.002	0.047	0.019	-0.058	-0.060	**0.107**	0.035	**0.156**

Other Psychological Problems	-0.028	**0.114**	0.031	0.025	-0.003	-0.032	0.025	0.002	-0.047	0.029	0.006	**0.069**
Medication for Mental Purposes	-0.026	**0.155**	-0.021	0.045	0.001	-0.073	-0.048	-0.023	0.029	0.047	0.036	**0.119**
Doctor Visits	0.036	**0.144**	**-0.059**	0.024	0.019	-0.065	-0.003	0.051	-0.046	0.044	-0.046	-0.005
Other Medication	**-0.060**	**0.185**	-0.038	**0.118**	0.001	0.067	0.032	-0.067	-0.046	0.054	0.006	**0.139**
Antibiotic Taken Past 3 Years	**0.066**	**0.099**	0.015	0.001	0.034	0.004	-0.010	-0.013	0.021	0.029	-0.016	**0.116**
Hospitalization_Past5Year	0.019	**0.124**	-0.002	-0.005	0.001	-0.062	0.047	0.001	0.023	-0.070	-0.027	**-0.058**
Life Expectancy	-0.013	**0.095**	0.012	0.036	-0.023	**-0.117**	-0.018	-0.015	0.066	-0.039	-0.021	-0.038
Now Feeling Physically Miserable	-0.007	0.073	-0.001	0.015	-0.007	0.017	-0.008	-0.010	-0.016	0.029	**0.073**	**0.102**
Usually Feeling Physically Miserable	-0.039	0.050	0.000	0.028	-0.047	-0.055	-0.046	-0.007	0.029	0.010	0.028	**0.169**
Now Feeling Mentally Miserable	-0.023	0.053	-0.006	-0.006	-0.028	-0.025	-0.019	0.010	0.024	-0.048	0.043	**0.128**
Usually Feeling Mentally Miserable	**-0.096**	0.034	-0.039	**0.072**	-0.013	**-0.151**	-0.045	**0.118**	-0.044	-0.006	0.023	**0.151**
Year	**0.406**	**0.082**	**0.379**	**-0.095**	**0.686**	**0.324**	**-0.419**	**-0.123**	0.050	**0.492**	-0.005	0.023
Course of COVID-19	NA	NA	0.004	-0.036	0.000	0.004	0.043	-0.033	-0.002	-0.003	**0.101**	0.050
Months since COVID Infection	**-0.179**	0.004	NA	0.017	0.037	**-0.405**	**0.469**	0.031	**-0.103**	**-0.479**	**0.059**	**0.059**

Sex (last column) was coded as 1 – men and 2 – women. Significant correlations are bolded. The p-values that remained or turned significant after the application of the Benjamini-Hochberg correction for multiple testing (with FDR set at 0.1) are underlined.

Table S23- P-values of the correlations between health and performance-related variables and COVID-related variables controlled for age, sex, survey year, and time elapsed since infection- All subjects

	Infected	Course	Months since Infection	Vaccination	Months since Vaccination	Covid after Vaccination	Ancestral SARS-CoV-2 variant	Alpha	Delta	Omicron	Age	Sex
Physical Health Issues	0.569	0.00	0.528	0.027	0.708	0.689	0.254	0.58	0.964	0.556	0.7	0.00
Mental Health Issues	0.053	0.101	0.427	0.081	0.671	0.33	0.445	0.651	0.527	0.437	0.177	0.00
Fatigue	0.209	0.021	0.107	0.002	0.341	0.86	0.006	0.017	0.19	0.703	0.77	0.001
Intelligence	0.598	0.162	0.194	0.125	0.855	0.004	0.089	0.094	0.8	0.00	0.498	0.001
Memory	0.17	0.812	0.144	0.176	0.801	0.946	0.016	0.454	0.175	0.266	0.06	0.00
Reactions	0.29	0.475	0.956	0.007	0.45	0.448	0.039	0.028	0.622	0.861	0.742	0.47
Accuracy	0.762	0.041	0.141	0.568	0.15	0.00	0.772	0.097	0.858	0.103	0.047	0.00
Output Variables												
Evolutionary Biology Score	0.985	0.83	0.007	0.377	0.075	0.024	0.746	0.246	0.75	0.384	0.00	0.005
Free-Recall Memory	0.131	0.673	0.088	0.919	0.751	0.65	0.107	0.377	0.304	0.512	0.098	0.00
Recognition Memory	0.089	0.653	0.076	0.695	0.83	0.605	0.108	0.112	0.098	0.544	0.181	0.00
Simple RT Duration	0.754	0.089	0.473	0.038	0.232	0.48	0.231	0.256	0.447	0.973	0.23	0.11
Simple RT Precision	0.746	0.823	0.747	0.071	0.222	0.619	0.486	0.00	0.134	0.237	0.768	0.007
Stroop Duration	0.55	0.32	0.8	0.066	0.729	0.795	0.087	0.109	0.654	0.25	0.984	0.921
Stroop Precision	0.954	0.019	0.34	0.679	0.218	0.048	0.195	0.35	0.1	0.378	0.53	0.733
Reading Time	0.185	0.776	0.385	0.083	0.773	0.264	0.381	0.214	0.77	0.876	0.982	0.05
Allergy	0.384	0.001	0.162	0.017	0.616	0.577	0.339	0.996	0.605	0.287	0.758	0.015
Skin Problem	0.568	0.00	0.992	0.57	0.439	0.052	0.762	0.207	0.466	0.964	0.281	0.523
Digestive Problem	0.838	0.002	0.657	0.022	0.257	0.328	0.785	0.351	0.334	0.715	0.338	0.855
Infection	0.00	0.00	0.308	0.294	0.651	0.358	0.121	0.004	0.697	0.342	0.041	0.054
Cardiovascular Problem	0.388	0.916	0.166	0.008	0.319	0.934	0.335	0.803	0.171	0.384	0.258	0.396
Low Blood Pressure	0.24	0.637	0.071	0.202	0.226	0.072	0.386	0.819	0.877	0.261	0.603	0.00
High Blood Pressure	0.153	0.999	0.66	0.453	0.079	0.022	0.229	0.918	0.151	0.996	0.034	0.384
Orthopedic Problem	0.333	0.249	0.884	0.086	0.506	0.915	0.955	0.014	0.531	0.108	0.097	0.00
Metabolic Problem	0.136	0.29	0.779	0.552	0.645	0.977	0.797	0.809	0.127	0.176	0.453	0.001
Neurologic Problem	0.059	0.002	0.072	0.137	0.442	0.18	0.681	0.191	0.796	0.468	0.589	0.003
Headache	0.999	0.001	0.311	0.00	0.498	0.737	0.906	0.292	0.65	0.379	0.109	0.00
Other Pain	0.566	0.00	0.452	0.866	0.898	0.811	0.109	0.727	0.964	0.234	0.291	0.002
Recurrent Problem	0.258	0.049	0.435	0.047	0.735	0.344	0.003	0.683	0.057	0.833	0.295	0.015
Depression	1	0.028	0.11	0.604	0.698	0.103	0.371	0.548	0.38	0.022	0.01	0.003
Anxiety	0.684	0.083	0.529	0.101	0.952	0.392	0.663	0.183	0.17	0.014	0.201	0.00
Other Psychological Problems	0.313	0.005	0.256	0.361	0.926	0.568	0.556	0.947	0.278	0.498	0.81	0.013

Medication for Mental Purposes	0.361	0.00	0.458	0.125	0.962	0.207	0.286	0.614	0.525	0.298	0.207	0.00
Doctor Visits	0.196	0.00	0.033	0.376	0.58	0.24	0.942	0.238	0.286	0.307	0.097	0.836
Other Medication	0.037	0.00	0.179	0.00	0.959	0.239	0.471	0.136	0.306	0.233	0.821	0.00
Antibiotic Taken Past 3 Years	0.017	0.014	0.572	0.957	0.333	0.943	0.812	0.753	0.624	0.502	0.551	0.00
Hospitalization Past 5 Years	0.493	0.002	0.93	0.85	0.961	0.264	0.276	0.977	0.585	0.107	0.319	0.035
Life Expectancy	0.629	0.019	0.652	0.2	0.509	0.036	0.675	0.731	0.13	0.365	0.449	0.17
Now Feeling Physically Miserable	0.787	0.066	0.945	0.575	0.841	0.76	0.846	0.819	0.706	0.497	0.008	0.00
Usually Feeling Physically Miserable	0.152	0.212	0.985	0.301	0.176	0.32	0.286	0.864	0.5	0.811	0.303	0.00
Now Feeling Mentally Miserable	0.401	0.186	0.811	0.826	0.422	0.643	0.661	0.819	0.583	0.265	0.116	0.00
Usually Feeling Mentally Miserable	0.001	0.388	0.153	0.01	0.693	0.007	0.303	0.007	0.313	0.873	0.391	0.00
Year	0.00	0.04	0.00	0.001	0.00	0.00	0.00	0.005	0.25	0.00	0.837	0.401
Course of COVID-19	NA	NA	0.909	0.364	0.991	0.941	0.33	0.449	0.964	0.931	0.012	0.212
Months since COVID Infection	0.00	0.909	NA	0.521	0.287	0.00	0.00	0.465	0.018	0.00	0.032	0.032

Figure S1- Changes in health and performance-related variables with time since COVID-related variables- Women

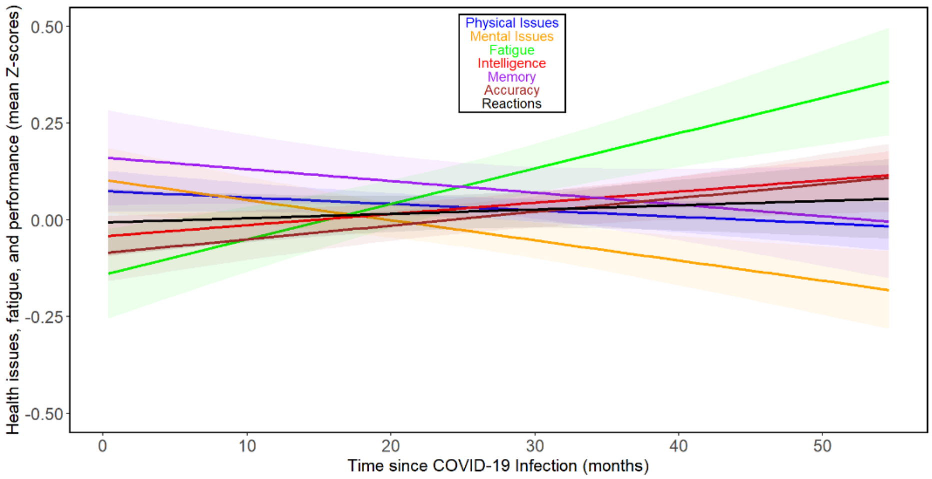

Based on the comparison of BIC values, all observed dependencies were best approximated by a first-degree polynomial (a straight line). The shaded areas around the lines indicate the 60% confidence intervals. It should be noted that the slopes of the lines on the graph cannot be directly compared with the (more accurate) Tau values calculated using the non-parametric partial Kendall test, which is less sensitive to the presence of outliers and additionally controlled for the influence of 3 confounding variables (sex, age, and year of survey).

Figure S2- Changes in health and performance-related variables with time since COVID-related variables- men

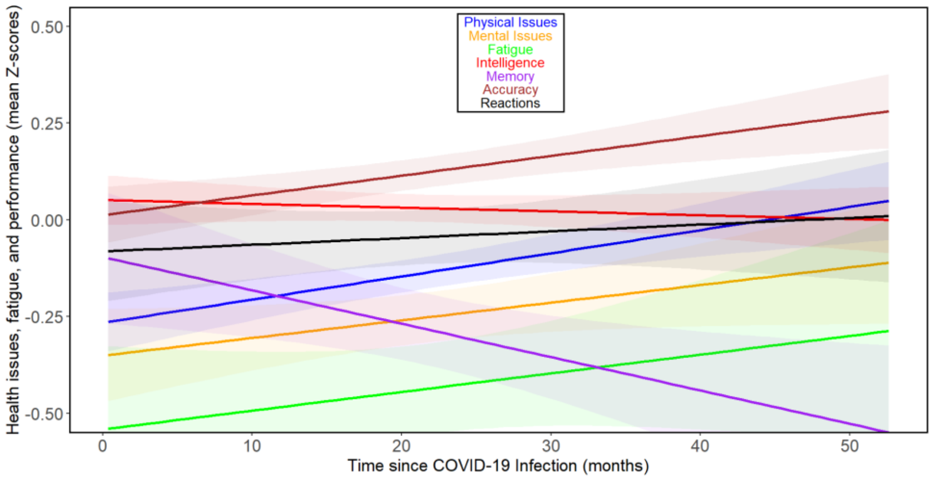

For legends see Figure S1.

Figure S3- Changes in health and performance-related variables with time since COVID-19 beyond 24 months post-infection- related variables- women

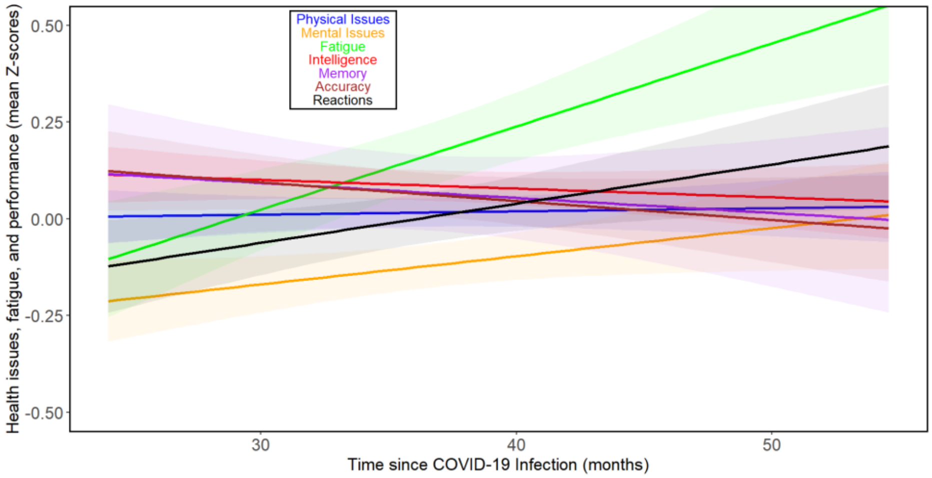

For legends see Figure S1.

Figure S4- Changes in health and performance-related variables with time since COVID-19 beyond 24 months post-infection- related variables- men

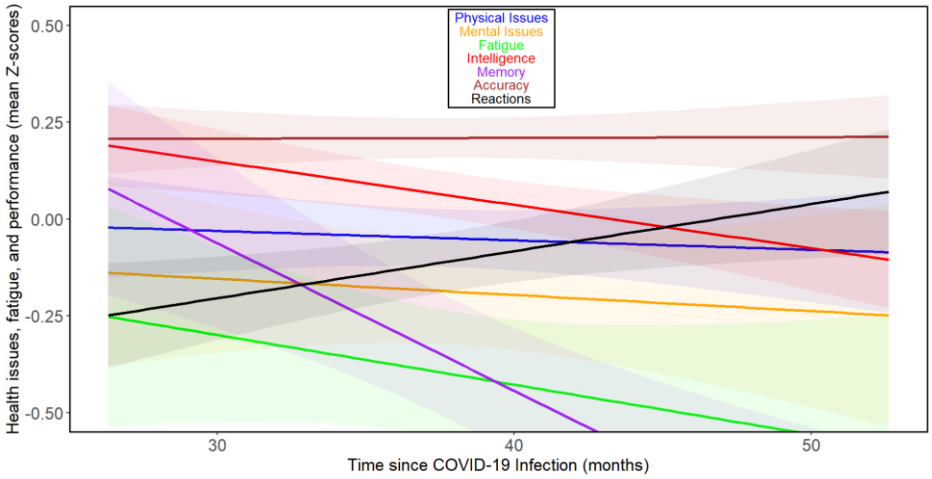

For legends see Figure S1.

Figure S5- Changes in health and performance-related variables with time since COVID vaccination-related variables- women

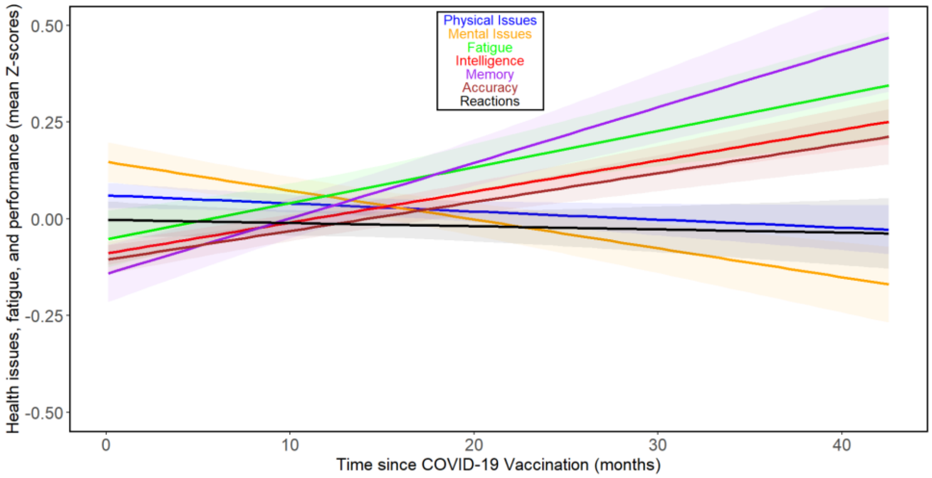

For legends see Figure S1.

Figure S6- Changes in health and performance-related variables with time since COVID vaccination-related variables- men

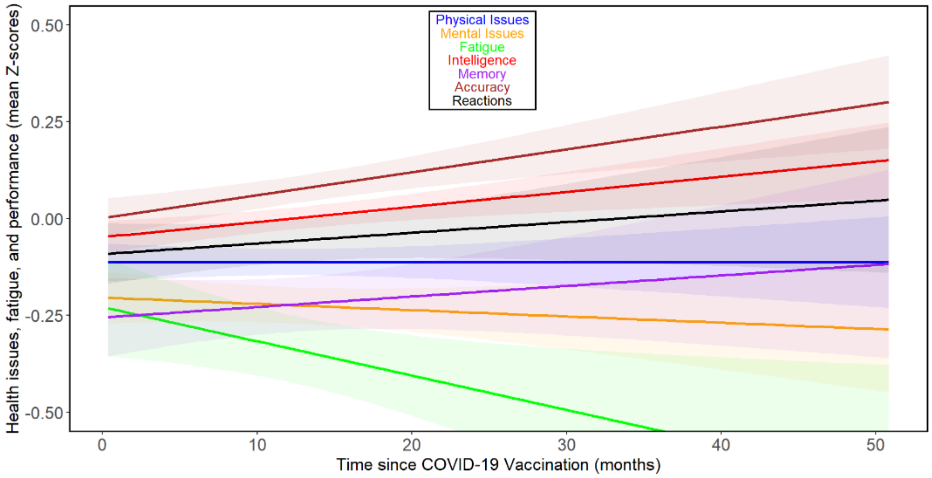

For legends see Figure S1.
